# Misinformation does not reduce trust in accurate search results, but warning banners may backfire

**DOI:** 10.1038/s41598-024-61645-8

**Published:** 2024-05-14

**Authors:** Sterling Williams-Ceci, Michael W. Macy, Mor Naaman

**Affiliations:** 1 Department of Information Science, Cornell University, Ithaca, NY USA; 2 Department of Sociology, Cornell University, Ithaca, NY USA; 3 Cornell Tech, New York, NY USA

**Keywords:** Trustworthiness evaluation, Search ranking, Misinformation/disinformation, Information reliability warnings, Search engines, Banner warnings, Human behaviour, Information technology

## Abstract

People rely on search engines for information in critical contexts, such as public health emergencies–but what makes people trust some search results more than others? Can search engines influence people’s levels of trust by controlling how information is presented? And, how does the presence of misinformation influence people’s trust? Research has identified both rank and the presence of misinformation as factors impacting people’s search behavior. Here, we extend these findings by measuring the effects of these factors, as well as misinformation warning banners, on the *perceived trustworthiness* of individual search results. We conducted three online experiments (N = 3196) using Covid-19-related queries, and found that although higher-ranked results are *clicked* more often, they are not more *trusted*. We also showed that misinformation does not damage trust in accurate results displayed below it. In contrast, while a warning about unreliable sources might decrease trust in misinformation, it significantly decreases trust in *accurate* information. This research alleviates some concerns about how people evaluate the credibility of information they find online, while revealing a potential backfire effect of one misinformation-prevention approach; namely, that banner warnings about source unreliability could lead to unexpected and nonoptimal outcomes in which people trust accurate information less.

## Introduction

Online searches provide an important source of information when people are making key critical decisions about their health and well-being^1–5^. Understanding how people evaluate the credibility of information in online search is critical because information from search engines can distort users' knowledge of topics^2,5,6^ and could potentially change their real-world behavior^7,8^. During the recent Covid-19 pandemic, for example, users turned to search engines for information about the disease and its treatment^9–11^. The reliance on search engines during a deadly pandemic has raised concerns about the potential threats to public health if people misjudge the trustworthiness of information they encounter in search results. But what, precisely, makes people trust some search results more than others? Can search engines influence people’s trust in information about critical health issues based on what information they show and how they present it? And, how does the presence of misinformation (and warnings about it) influence people’s trust in search engines’ results?

We address these overarching themes in a series of online experiments evaluating specific research questions focusing on people’s trust in search results, particularly concerning reactions to the ranking of the results, the presence of misinformation, and the addition of misinformation warning banners on search pages. To this end, we performed three online experiments with a total of 3196 participants measuring people’s evaluations of the trustworthiness of search-results’ sources in the context of Covid-19. Participants were asked to click one result that they would choose to answer the query, then to rate one semi-randomly selected result from the page on a scale of perceived trustworthiness. Across all experiments, we randomized the rank of results (their order on the page) and manipulated whether misinformation was present in a high-ranked result; in Experiment 3, we additionally tested whether the inclusion of different warning banners impacted trustworthiness evaluations. The experiments used different Covid-19 queries varying in the degree of consensus at the time of the study, attributed the results to two different search engines (Google and DuckDuckGo), and used search results with both familiar and unfamiliar sources.

Our results were consistent: Despite finding support for the well-established rank-click relationship, we found no parallel relationship between rank and trustworthiness evaluations for accurate information. Further, we showed that misinformation was highly distrusted in comparison to accurate information, even when shown at or near the top of the results list. Additionally, we showed that the presence of high-ranked misinformation does *not* harm the perceived trustworthiness of accurate results below it. However, a warning banner about the unreliability of the results’ sources showed a possible backfire effect: one of the banners we tested caused a decrease in the perceived trustworthiness of accurate results, although we could not establish whether it had the same impact on misinformation results on the page. The contributions of this research are threefold: (1) Our results assuage common fears regarding search engines skewing people’s trust toward misinformation. (2) Our findings reveal unforeseen effects of vague misinformation warnings similar to those currently being shown by Google. Lastly, (3) Our research illustrates implications for the design of interventions to guide people’s trust toward accurate information in online searches.

### Background

Research across disciplines has examined (a) the impact of rank on people’s decisions to click search results, (b) the cognitive impacts of misinformation in online systems, and (c) fact-checking strategies to combat misinformation. Our current study connects these factors by studying their joint impacts on how 3196 people assessed the trustworthiness of high-stakes medical information provided by search engines.

#### Impact of rank in search

Numerous studies have found that the rank of results in search is correlated with their probability of being clicked^5,12–16^. Several explanations have been proposed, many of which relate to Cognitive Miser theory^17^. This theory proposes that humans avoid using mental resources when making decisions, which leads them to rely on heuristics or cognitive short-cuts. For example, Azzopardi^18^ describes clicking on high-ranked results as a “satisficing” behavior by cognitive misers, allowing them to avoid scrolling through countless results and considering an overwhelming amount of information (p. 32). In support of this hypothesis, Pan and coauthors found that people are more likely to click on high-ranked results even when these results were independently judged as being the least relevant in the list^14^.

While the cognitive miser hypothesis is widely accepted, an additional factor may play a role in the decision to click high-ranked results: people may interpret high rank as an indication that the content is trustworthy. Research on Cognitive Miser Theory predicts that people abandon heuristics and turn to more rigorous analysis of information’s trustworthiness when faced with high-stakes situations that demand accurate decisions^17,19^, of which medical questions are a prime example. Nevertheless, studies have shown that people still rely on rank when deciding which results to click in response to health-related queries^5,14–16^. One possible explanation for this finding is that people assume a result’s rank is indicative of its trustworthiness. This could be concerning if true, because individuals searching for health information may be especially vulnerable to one of two outcomes: trusting high-ranked misinformation and/or distrusting accurate information ranked below it.

Despite ample research on how people evaluate trustworthiness of information in online search, the relationship between rank and trustworthiness evaluations has not been studied directly for multiple reasons. For one, studies have relied on click-rates as a proxy, instead of directly measuring perceptions of trustworthiness^12,14,15,20^. For example, Pan et al.^14^ and Schultheis et al.^20^ found that click probability was correlated with the ranking of results more than with their true relevance to the query, but they did not measure whether participants also *trusted* higher-ranked information more than the information that was ranked lower (Pan et al.’s paper was misleadingly titled “In Google We Trust,” but referred to the finding that people trust Google’s ranking to provide the most *relevant* results first). Haas and Unkel^12^ more directly examined how people evaluate the credibility of individual search results by asking participants to rate individual results on credibility scales, and found impacts of source credibility cues in the results’ headlines on participants’ credibility ratings thereof. However, the researchers did not manipulate the results’ rank when they had participants do the actual trustworthiness evaluation. In their words: “When rating the credibility of the search results, participants in our study only saw each result by itself. The results were not displayed as part of the SERP [search engine result page]. Therefore, it remains unclear if the perception of credibility is also affected by the ranking […]” (p. 22).

Given this gap, we were interested in formally testing whether rank impacts trustworthiness evaluation as well as click likelihood. Since the rank-click relationship has been documented in numerous studies, including for health-related queries, we first propose this relationship as a baseline hypothesis:

##### H0

An increase in the rank of a search result will be associated with an increase in the probability of it being clicked.

Prior work has shown the importance of trust when selecting information in high-stakes contexts ^5,21,22^. Therefore, we hypothesize that people will trust results they choose to click more than results they do not click.

##### H1

Controlling for rank, participants will rate the trustworthiness of results they clicked higher than ones not clicked.

However, the central question is whether people *trust* high-ranked results more than lower-ranked ones, which has been assumed but not directly tested in prior work ^4,7^. Given the consistent rank-click relationship when searching for medical advice (despite the high stakes for personal wellbeing), we propose a second form of Cognitive Miser behavior in which people interpret rank as a signal of results’ trustworthiness:

##### H2

An increase in the rank of a search result will be associated with the result being rated as more trustworthy.

#### Impact of misinformation in trustworthiness evaluation

If people believe that high-ranked results hold the most trustworthy information, misinformation becomes especially concerning. Search engine audits have found that misinformation appears in search results with alarming frequency^3,23^, including in high-ranked results^24^. The prominence of misinformation in search engines carries especially significant consequences for public health challenges such as Covid-19: Recent studies have found that exposure to false or misleading search results is associated with an increased likelihood of ill-advised medical decisions^2,5,22^, with Swire-Thompson and Lazer^25^ concluding that “Misinformation concerning health has particularly severe consequences with regard to people's quality of life and even their risk of mortality” (p. 443).

The problem may go beyond the willingness to believe misinformation^10^. While some studies have found that people are good at identifying blatant misinformation^26^, researchers have warned that exposure to prominently displayed misinformation may undermine trust in subsequent *accurate* information^2,27,28^. Song and Jiang^2^ found that exposure to a higher number of search results with misinformation was associated with a decreased likelihood of clicking on accurate results in the list and a higher rate of inaccurate medical decisions. These studies show the potential impact of misinformation in search engines, and its interactions with accurate information in influencing people's knowledge. However, it is still an open question as to whether the mere presence of misinformation in a high-ranked result causes people to distrust lower-ranked accurate information in search engines.

We address this research gap by manipulating whether misinformation appears in high-ranked search results. We first test whether people recognize when a result contains misinformation. We hypothesize that participants in our main studies will click our misinformation results less often and rate them as less trustworthy than accurate results in the same rank:

##### H3a

A result containing misinformation will be less likely to be clicked than a result containing accurate information, controlling for rank.

##### H3b

A result containing misinformation will be trusted less than a result containing accurate information, controlling for rank.

Assuming participants recognize misinformation in search results, does it indeed cause them to doubt the trustworthiness of accurate information presented below? Given the evidence above, and given that exposure is guaranteed when misinformation is shown in a high-ranked search result since people scan search pages from top to bottom^29^, we hypothesize that:

##### H4a

An accurate result will be less likely to be clicked when the result immediately above it contains misinformation, compared to the control condition in which only accurate results are shown.

##### H4b

An accurate result will be rated as less trustworthy when the result immediately above it contains misinformation, compared to the control condition in which only accurate results are shown.

#### Impact of warning banners in trustworthiness evaluation

**Figure 1:**
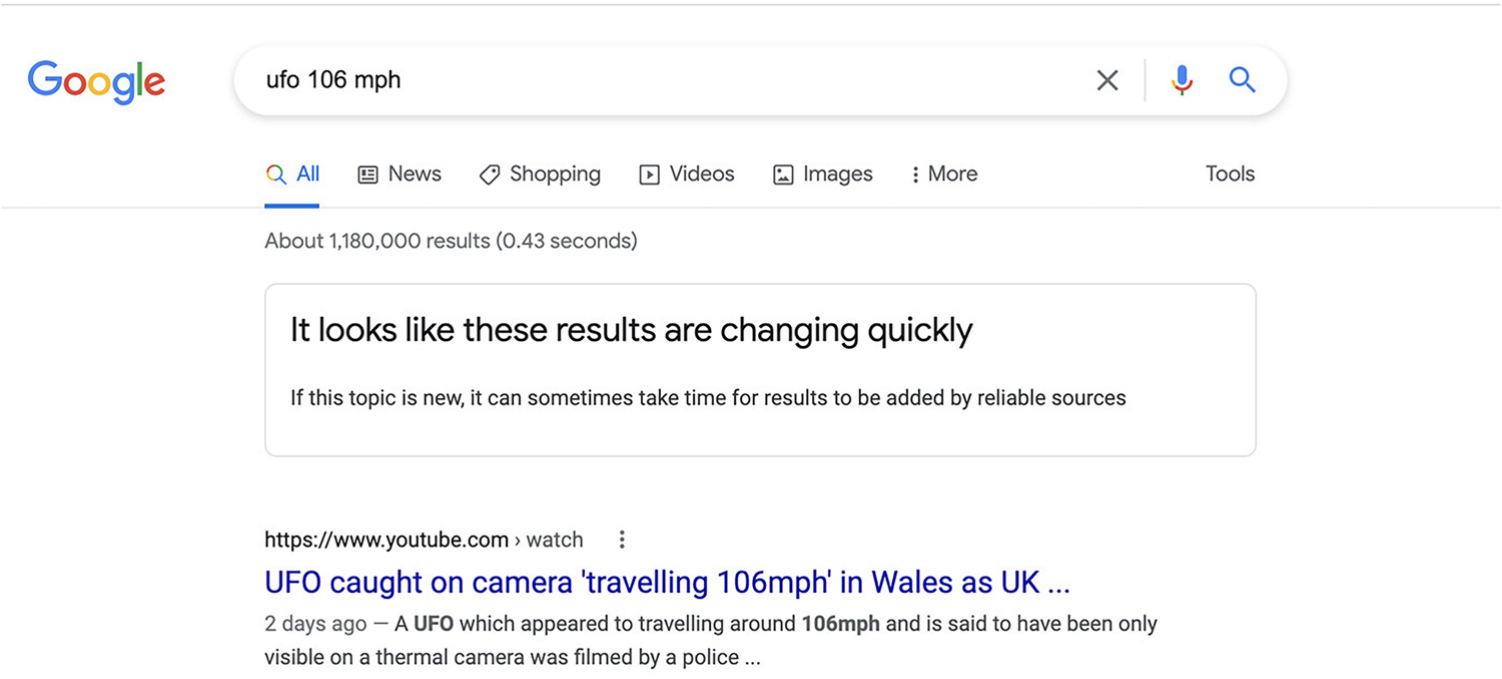
A Google warning banner for unreliable information in search results^22^.

Concerns about misinformation in search results have led Google to place warning banners at the tops of pages with unreliable search results. Figure 1 shows an example provided in a Google blog post, which says these warnings are intended to help people “confidently evaluate the information [they] find online”^30^. The initiative has been praised as a promising step toward protecting people from unreliable information^31^.

These banner-style warnings are distinct from traditional misinformation warning labels attached to specific pieces of content in social media, news feeds, and even search engines^32^. Item-specific warnings have been the subject of extensive prior literature with conflicting findings^32,33^. For instance, Pennycook and colleagues^33^ found that people who saw a list of headlines, with warning labels attached to some, automatically assumed the headlines without these labels were accurate–the “Implied Truth Effect.”

Research has also begun to address the effects of general information reliability warnings, albeit in contexts other than search engines, and with similarly-mixed findings. Roozenbeek et al. found that YouTube videos describing hallmarks of misinformation improved viewers’ ability to discern accurate content from inaccurate content^34^. Epstein et al. did a search-engine-specific study with similar warning banners about search results being biased: they found that these alerts can mitigate the impact of biased search result pages on how much people trust political candidates who are being portrayed^8^. However, other studies have found null effects of the general warning approach^35^ and, more alarmingly, that these warnings can harm credibility perceptions of *accurate* news presented afterward^27,36^. Of these studies, Epstein et al.^8^ was the only one to test the impacts of warning banners in search pages, and none has looked at how these general warning banners impact trustworthiness evaluations of individual search results about a topic.

These studies show the importance of understanding how warning banners affect people’s trustworthiness evaluations of both true and false information in search results, and whether these banners moderate the possible effects of rank and misinformation on click behavior and trust. Based on these studies of general warnings from other contexts, we propose that:

##### H5

Ratings of results’ trustworthiness by those who see a warning banner will be lower than the ratings by those who see no warning.

## Methods

We used three online experiments to address the hypotheses listed above. The experiments were somewhat different in their design, though each had the same basic structure, in which participants interacted with a search engine result page and were then asked to evaluate the trustworthiness of one or more of the results from the list.

This research was approved and exempted from full review by Cornell University’s IRB office (protocol #0010181), and informed consent was obtained from all participants prior to data collection. All experiments were performed in accordance with relevant ethics guidelines and recommendations. All hypotheses and analyses were pre-registered on AsPredicted.com.

### Procedure

Our experimental setup across the three experiments involved showing online participants three search engine result pages (SERPs) in random order. We designed these pages to look like real pages of Google search results, with a query shown in a search bar and ten search results listed below it. These search results were the traditional “blue link” type of search results; no Knowledge Panels or other features available on Google were shown. In each experiment, two of these pages were distractors with queries about other topics, which we did not analyze. The remaining search result page was the setting for each experiment: it had a query about Covid-19 selected at random from three different queries for each experiment, and a set of associated results shown in random order. The main factors of the Covid-19 search pages that we manipulated in both experiments were the order of the results with accurate information, and the presence and location of a result with misinformation. A control group saw ten accurate results presented in Google’s interface, while treatment groups saw the same page but with one of the top three accurate results replaced by misinformation.

We collected the accurate results for each experiment by issuing the query on Google, then selecting results that came from established medical websites which agreed with the scientific consensus about the query at the time. The misinformation results shown were either gathered from Google or created by the researchers, and were pretested to confirm our judgments of falsehood, as described below. All the queries, including the decoy queries, and their search results are included in the Supplementary Information.

To avoid potential effects of a single query, a specific misinformation result, or a fixed ranking of the accurate results, we randomized all these factors. First, we randomly assigned participants in each experiment to see one of the experiment's three distinct queries about Covid-19. For the conditions in which misinformation was shown, we randomly selected one of the three misinformation results specific to that query and showed this result in the designated rank. Lastly, we randomized the order of the accurate results in every condition across all experiments.

**Figure 2:**
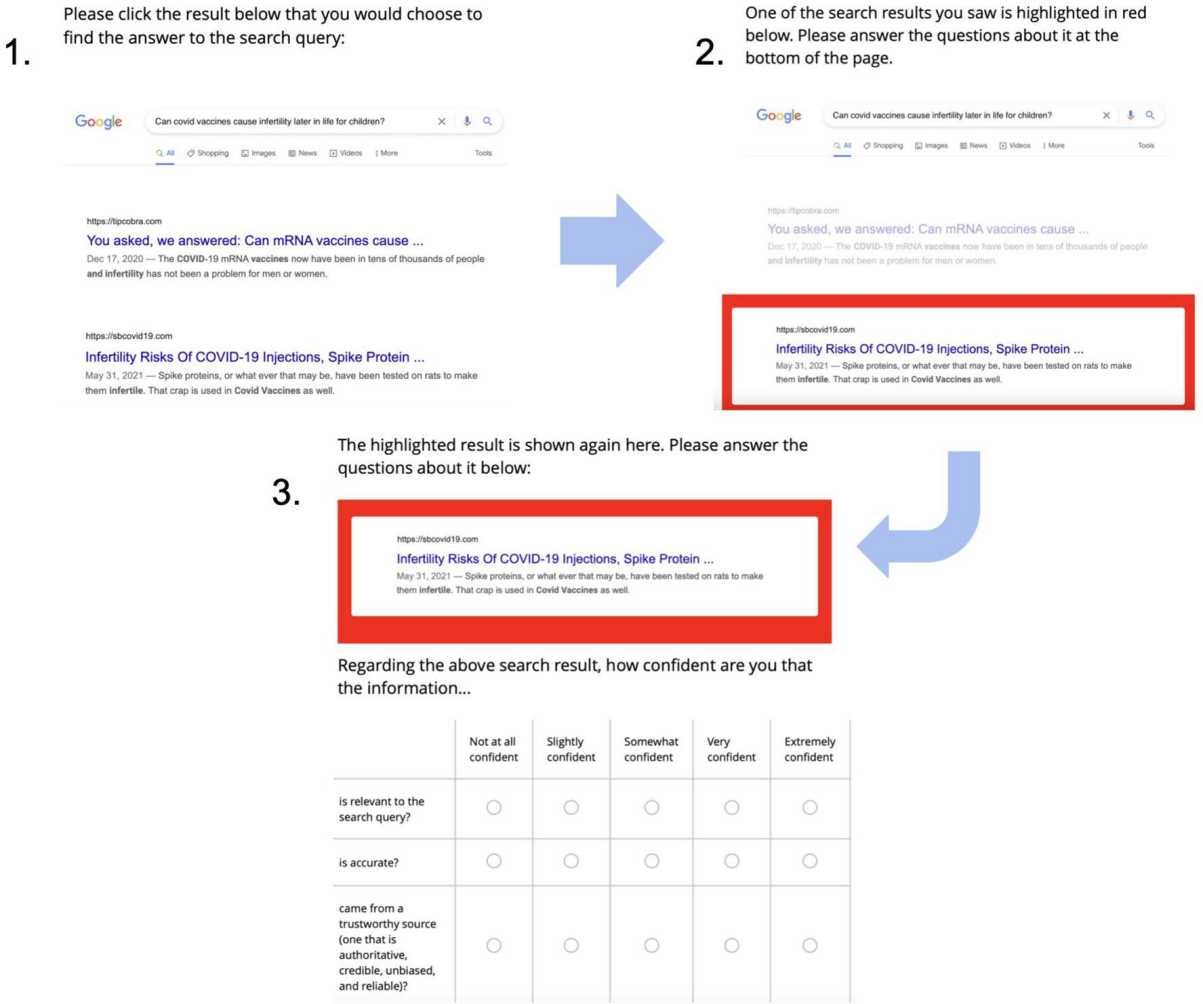
The survey interface developed for the three experiments. This figure shows an example of a page seen in Experiment 2 in the condition with misinformation in the second rank. (Note: there were ten total results shown on the first page that could not all be captured in a screenshot. We ensured that participants saw all ten results by making them scroll to the bottom of the page to continue the survey).

For each experimental query, we measured click decisions by asking participants to click on one result that they would choose to answer the query in question. After participants clicked a search result, we showed the page of results again, with one of the results highlighted in red and the rest of the results grayed out. We showed the highlighted result again at the bottom of the page, where we asked participants to indicate their opinions of the result’s trustworthiness (see “Measures” for details). The reason we showed the result in the original page context was to subtly remind the participants of its rank on the page. In each experiment, the result(s) participants rated were chosen randomly after a balancing procedure to ensure we would have ratings for clicked and non-clicked results, as well as accurate and misinformation results. We also collected participants’ ratings of the result’s accuracy and relevance, which we only used to check their correlations with participants’ trustworthiness ratings. After rating the result(s), participants were directed to answer optional questions about their relevant attitudes and demographics (see “Measures”). The survey procedure is illustrated in Fig. 2.

We used Prolific Academic for recruitment in each study. We screened for participants who resided in the U.S. (due to the unique relevance of the Covid-19 vaccine queries to this population at the time of the study) and who were fluent in English (due to the search results all being in English). We also requested to get a sample evenly split between women and men. Before starting the experiment, participants were asked to give their informed consent and explicitly confirm that they were using a desktop or laptop computer (which was necessary for the survey to work properly).

At the end of the survey, we showed participants a debriefing statement before we confirmed their compensation. The statement disclosed that they may have seen information with harmful misinformation about Covid-19, and that they should not take the information from our search results as medical advice.

### Experiment 1

**Figure 3:**
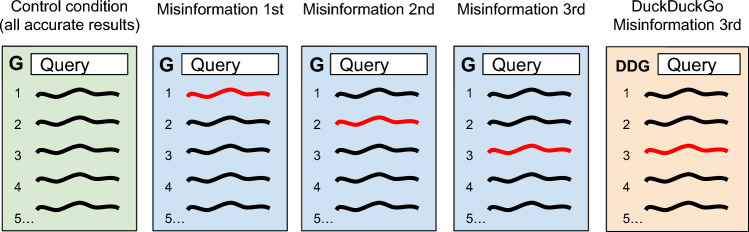
Conceptual illustration of the conditions in the between-subjects experimental design used in Experiment 1 and Experiment 2.

Experiment 1 (deployed in January 2022) used five between-subject conditions, alternating the presence and rank of the misinformation result and the search engine the results were associated with. The experiment design is illustrated in Fig. 3. A control group showed all accurate results and displayed the search results page as if created by Google. The four treatment conditions showed misinformation ranked first, second, or third in Google, or ranked third in DuckDuckGo.

We used queries and associated results on the topic of the widely discussed safety of Covid-19 vaccines for children. The three queries we used for this experiment represented child-specific concerns about the vaccines’ safety, potential long-term risks to fertility, and potential cancer-causing effects. These concerns have been documented frequently in search logs^1^. Although the topic remains politically controversial, the FDA had authorized the vaccines for use in children starting at age five and multiple peer-reviewed scientific reports supported their safety in this age group^37,38^. This data provided a reliable benchmark for identifying medical misinformation.

We sourced misinformation results for our treatment groups in Experiment 1 by searching for each of the three queries about Covid-19 vaccine safety on Google and collecting results that (1) came from social media sites and (2) had extreme claims that the vaccines were unsafe. We validated our choices of misinformation results by running a pretest in which we showed pages of ten search results to a sample of individuals from varying socioeconomic and political backgrounds (N = 30). Five of these results had information that contradicted the scientific evidence showing Covid-19 vaccines were safe for children. We randomized the results’ order for each participant and asked them to select any results that they believed had false information. We chose the three results that were most frequently chosen as misinformation to be used in experiments 1 and 2. The Supplementary Information provides additional details about the pretest.

Participants in Experiment 1 were asked to indicate the perceived trustworthiness of three results on the search page (one of which was the result they had clicked). We randomly selected two non-clicked results from the top five results shown to the participant, due to past studies indicating that people pay the most attention to search results in this range^29^. Importantly, for participants in the misinformation treatment conditions, one of the results they were asked to evaluate was always the misinformation result, regardless of whether they clicked it. This design allowed us to measure participants’ levels of trust in a range of results: those clicked by participants, those which were not clicked, and results that were accurate information or misinformation.

We recruited 1000 participants (200 per condition) to be able to detect a small effect size of f = 0.1 for the effect of rank on trust in accurate results controlling for click status, at 80% statistical power (the Supplementary Information provides additional details on this analysis). After collecting this sample, we excluded data from one participant who had a missing value for their unique ID and one who failed our attention check, leaving 998 participants and 2,994 trustworthiness evaluations for analysis.

Our final sample for Experiment 1 consisted of young adults of average age of 37.2 years (SD = 13.4); 47.9% identified as women. Participants were left-leaning (66% identified as leaning liberal, 26% leaned conservative, and 8% had no opinion), supportive of vaccination (85% of participants indicated support for vaccination, 7% indicated non-support, and 8% indicated having no opinion). We discuss the challenges presented by the skew of this sample in the "Discussion”, and we note demographic-specific effects of all findings in the Supplementary Information. Participants were generally trusting of the search engine they were shown (for Google, 78% indicated trusting the search engine while 16% distrusted it and 7% indicated having no opinion; for DuckDuckGo, 79% indicated trusting the search engine and the remaining participants indicated having no opinion).

### Experiment 2

Experiment 2 (deployed in April 2022) used the design of Experiment 1, but expanded on that study by testing the effects of rank and misinformation on trustworthiness when participants were unfamiliar with the sources of the search results. To this end, we obtained eighteen candidate source URLs from real Google search results for the same query as Experiment 1. We then ran a pretest using a separate group of Prolific participants (N = 66) to validate their unfamiliarity with these sources. We asked participants whether they recognized each website and how realistic each website sounded (to ensure that the sites we used would still seem plausible to participants in the main studies; the Supplementary Information provides additional details). We ended up with 13 sources that had at least 87.5% of participants who failed to definitively recognize them. We used the same search results’ text body from Experiment 1, but we randomly assigned one of the less-known sources to be shown above each of these results’ text instead of their original sources. We saw evidence that participants noticed the uncertainty of the sources in that the average trustworthiness rating given to accurate results in this experiment ($\underline{x}$ = 2.81 scale points) was significantly lower than in Experiment 1 ($\underline{x}$ = 3.41 scale points), as seen in a t-test (*t* = 12.8, *p* < 0.001; though we note that formally comparing these means via a statistical test is misleading, since the experiments used different samples of people from different time points).

In addition to changing the sources to be unfamiliar, we changed the procedure from Experiment 1 to Experiment 2 in three ways: (a) we revised the trustworthiness rating prompt to be directed at the results’ sources (see “Measures” for details on this change); (b) we asked participants to rate only *one* of the search results because we did not want to prime participants to pay extra attention to the manipulation of result rank, which could engender unnatural responses due to demand characteristics; and (c) we expanded the range of non-clicked results they could be assigned to rate to all results that appeared on the page to do a more comprehensive test for the hypothesized rank-trust relationship.

We again recruited 1000 individuals, and excluded two from analyses who failed our attention check, leaving a final sample of N = 998 participants. Our sample had similar characteristics to that of Experiment 1 (mean age = 40.2 years, SD age = 14.6 years, 48.5% women).

### Experiment 3

We ran a third experiment (deployed in August 2022) to test how the outcomes from the previous experiment might change when people are faced with banner-style warnings about potential misinformation and uncertainty about the queries’ answers in addition to the sources’ reliability.

We replaced the vaccine safety queries from the first two experiments with three new Covid-19 queries that lacked medical consensus at the time of the study. We obtained uncertain queries by searching for questions in the news about Covid-19 and found nine that had at least two conflicting answers on the first result page, each endorsed by medical sources. We then conducted a pretest to confirm Prolific participants were uncertain about these queries. The pretest identified three queries, used in this experiment, for topics that participants were most split about: the possibility of animal-based transmission, the value of double-masking, and the efficacy of natural immunity compared to vaccine-induced immunity (for more pretest details, see the Supplementary Information). We saw evidence that participants noticed the uncertainty of the queries, in that the average trustworthiness rating given to accurate results in this experiment ($\underline{x}$ = 2.55 scale points) was significantly lower than in Experiment 2 ($\underline{x}$ = 2.81 scale points) as seen through a *t*-test (*t* = 4.8, *p* < 0.001; though we note that formally comparing these means via a statistical test is misleading, since the experiments used different samples of people from different time points).

For each of these queries, we found ten authoritative search results from Google (defined as those having information supported by multiple medical sources). Due to the uncertainty of the queries, we were unable to definitively classify any of the Google results as misinformation. Therefore, we created several fake results with blatant false claims about the pandemic in general (e.g. “Covid is a hoax”). To ensure that people perceived these results as being false but still realistic, we did another pretest on a separate group of Prolific participants (N = 80) in which we asked them to rate the misinformation results we created and some of the accurate results on perceived accuracy and perceived likelihood of appearing online (see the Supplementary Information for details). We ultimately selected three misinformation results for each query that had significantly lower accuracy ratings and were statistically indistinguishable in perceived likelihood of appearing online. Finally, when displaying the result pages, we randomly paired each search result’s text snippet with one of the unknown sources like in the previous experiment.

**Figure 4:**
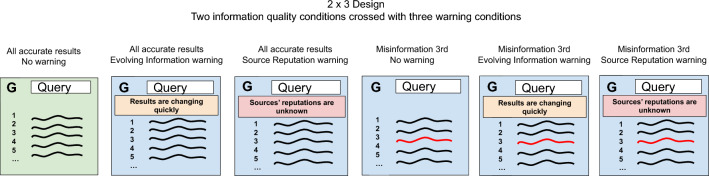
Conceptual illustration of the conditions in the between-subjects experimental design used in Experiment 3.

We made three changes to the experimental design for this study. Instead of showing a misinformation result in one of the top three ranks, we only showed it in the third rank because misinformation’s exact rank was not a moderator of any effects seen in the previous studies. For the same reason, we omitted the DuckDuckGo condition from this study. Most importantly, we added a new between-subjects manipulation in which we displayed one of two possible warning banners, or no banner, at the top of the search page, to test the effect of banners on the evaluation of results. This two-by-three design is illustrated in Fig. 4.

**Figure 5:**
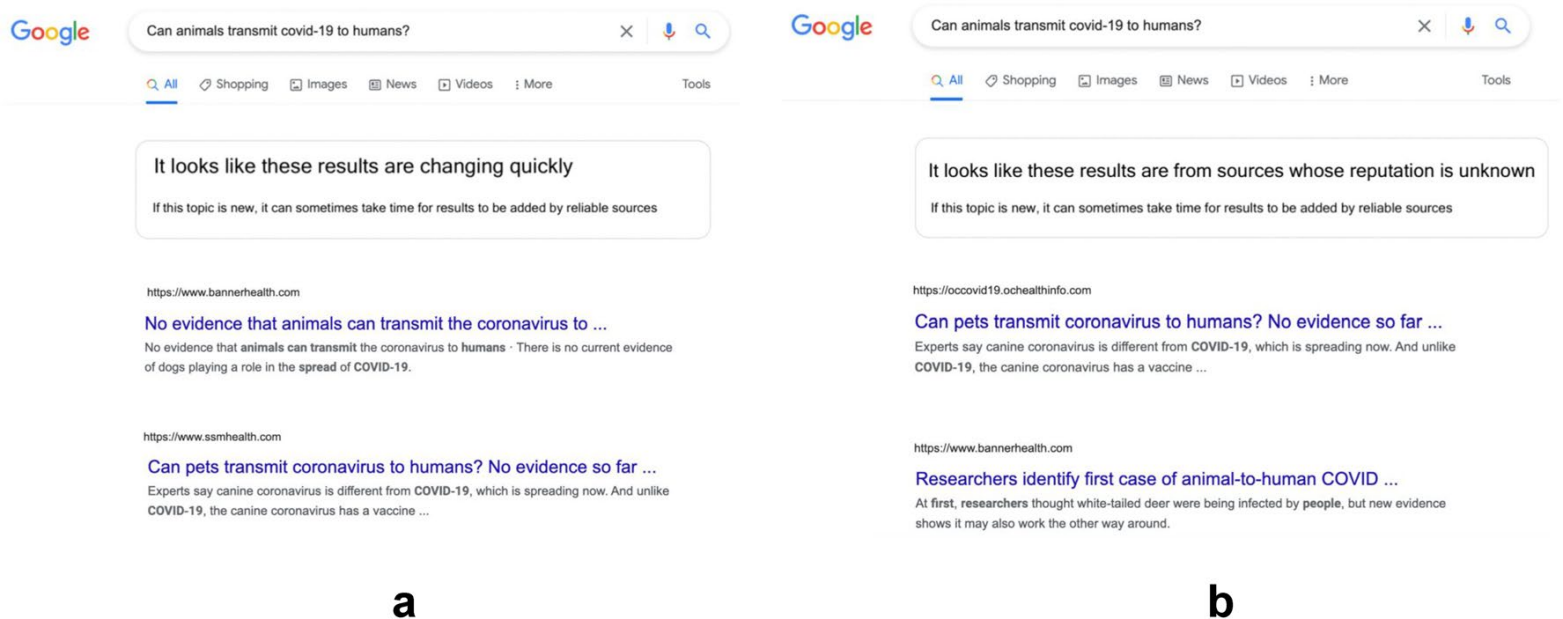
The two types of warning banners in Experiment 3. On the left (**a**) is Google’s “evolving information” warning. On the right (**b**) is the “source reputation” warning we created.

One of the banners we implemented, which is currently used by Google^30,31^, mentions that the results are changing quickly with subtext telling participants that it can take time for results from reliable sources to appear (the “evolving information” warning). The second banner has the same subtext as the “evolving information” warning, but its main text instead says that the sources' reputations are unknown. This “source reputation” warning tests whether alerting participants to the unreliability of the search results’ sources has different effects on trustworthiness evaluations than more vaguely warning them about the information changing quickly. The two banners are shown, as they were displayed to participants, in Fig. 5.

We recruited 1200 participants (200 per condition as recommended by the power analysis). We included all participants' data in our analyses because none failed our attention check. This experiment's sample closely resembled that of the previous experiment with a mean age of 36.2 years (SD = 12.9) and 48.8% identifying as women.

### Measures

Our hypotheses test the potential effects of rank, misinformation presence, and misinformation warning banners on our two main dependent measures: click behavior and perceived trustworthiness.

Participants were first asked to click a result on the search page with these instructions: “Please click the result that you would choose to find out the answer to the question being searched.” Participants were only allowed to click one result, and we recorded the rank of this result as well as whether it contained accurate information or misinformation.

Our main outcome of interest was how trustworthy people perceive individual search results to be. Perceived trustworthiness has traditionally been framed in terms of perceptions of ability, benevolence, and integrity of the trustee in interpersonal contexts^39^; this work has been extended specifically in online information contexts to include more specific constructs, such as perceived authoritativeness and objectivity of the source^40^. However, there is a lack of consensus among past studies on how exactly to measure perceived trustworthiness of online information^41^: some studies have used single scales that generally ask about the extent to which the information is trustworthy^15,19^, whereas others have used multiple scales that form a composite measure of perceived trustworthiness directed at both the content and the source^40^. We had to make a decision about which of these approaches to take when asking participants to evaluate search results in our experiments.

When we reviewed these studies, we noted that the multi-item trustworthiness measures had high inter-item correlations^12,42^. We also found this to be true in our own pilot study, in which responses to multiple facets of trustworthiness loaded highly onto a single factor and had a high degree of consistency (all measures’ loadings greater than or equal to 0.81, N = 97, Cronbach’s alpha = 0.93; see the Supplementary Materials’ Sect. 5 for details). These factors – along with the risk of inducing fatigue from having participants rate each search result on repetitive scales – led us to choose simplified prompts similar to those in past work^15,19^ for measuring perceived trustworthiness of the results in each experiment.

In Experiment 1, we had participants rate how trustworthy they perceived the result(s) to be by asking them “How confident are you that the result is trustworthy?” This prompt is very similar to that used by Salmerón, Kammerer, and García-Carrión^19^, who measured how people evaluate the perceived trustworthiness of individual web pages.

Upon finding that accuracy and trust ratings were highly correlated in Experiment 1 (*r* = 0.93), we modified this measure to more clearly define trustworthiness in the following experiments: “How confident are you that the information came from a trustworthy source (one that is authoritative, credible, unbiased, and reliable)?” Metzger and Flanagin found that people think about these aspects of sources as heuristics when determining how trustworthy online content is, and that they often think about more than one of these attributes at once^40^. By mentioning these trustworthiness heuristics within the prompt, we aimed to disambiguate trustworthiness from accuracy and other constructs in participants’ minds. We found that adding these terms decreased the correlation between accuracy and trustworthiness ratings (from *r* = 0.93 in Experiment 1 to *r* = 0.81 in Experiment 2 and *r* = 0.84 in Experiment 3), suggesting that this revision helped differentiate these factors. We note, however, that the basic findings were consistent in each experiment despite this change, which supports the robustness and validity of our findings.

Participants responded using a Likert scale of confidence (1 = “Not at all confident”, 5 = “Extremely confident”). This scale has been used to measure the strength of people’s attitudes in uncertain contexts^43,44^, including attitudes about the trustworthiness of Covid-19 information^45^.

To better understand the demographic and attitudinal composition of our samples, we measured the following variables in a post-task survey: trust in the search engine shown in the study ^39,42^, support for vaccination^46^, political ideology^47^, age, and gender. The Supplementary Information provides breakdowns of these variables for each sample and any moderation effects they had on our manipulations.

### Analytic approach

We used logistic regression models to analyze the effects of rank on click behavior (H0) and to investigate the moderation effect of the warning conditions on this relationship. For testing the rank-trust relationship (H2), we used linear regression models that controlled for whether participants had clicked the result (a necessary control due to limiting the range of non-clicked results that we assigned participants to rate in Experiment 1). To test the impact of misinformation on the likelihood of clicking a result in one of the top three ranks (H3a), we used Chi-Square tests. To analyze the association of clicking on people’s trust ratings of the results themselves (H1), as well as the effects of information quality (H3b) and warnings (H5), we used ANOVA tests (repeated-measures ANOVAs for Experiment 1 were used to test effects of clicking and information quality, since we had participants rate multiple results that varied in these factors). To test the impact of misinformation’s presence on people’s propensity to click (H4a) and trust (H4b) accurate results immediately below it, we used Chi-Square tests and ANOVAs, respectively. Our models generally had normal residuals and homoscedasticity of variance; we also confirmed our findings with non-parametric tests.

We used the Type III Sums of Squares ANOVA to test for interactions where preregistered; if no interactions were significant, we reran the model using the Type II setting to test for main effects. To decompose significant main effects and interactions, we used pairwise comparisons of estimated marginal means for categorical predictors, and simple slopes analysis for continuous predictors, applying Bonferroni adjustments for multiple contrasts. All statistical tests were two-tailed. All analyses were done with RStudio version 2022.07.0 and are reproducible using the data and code in the OSF repository for this project.

The analyses presented below deviate from the preregistered approaches in minor ways. First, we preregistered using a linear regression to model the effect of rank on results' click probability, but later corrected the approach to using a logistic regression model as logistic regression is more appropriate for a binary outcome. Second, we preregistered models testing for 3-way interactions between results' rank, click status, and information accuracy when predicting trustworthiness ratings. Because there was an unexpectedly low number of participants who clicked on the misinformation result, we lacked statistical power to detect this type of interaction if it existed; thus, we report on models in which we test whether rank interacts with click status or accuracy individually. Importantly, none of the findings changed when running the preregistered analyses, except for one secondary analysis on differences in the strength of the rank-click relationship depending on warning condition (see “Responses to Warnings” in the “Results”). All preregistered models are reported in the Supplementary Information.

## Results

We present the analyses for click behavior and trustworthiness judgments in response to the three main factors we manipulated: result rank, the presence of misinformation, and the inclusion of a warning banner about unreliable information or sources. Due to the experiments’ similarity and the increased statistical power gained from aggregating their data, we report on estimates for all the data in addition to separate estimates from each individual experiment. We followed these confirmatory analyses with exploratory models that tested for interactions between each manipulation with demographic factors and found some demographic-specific results; please see the Supplementary Information (Sect. 9) for details.

### Responses to rank

**Figure 6:**
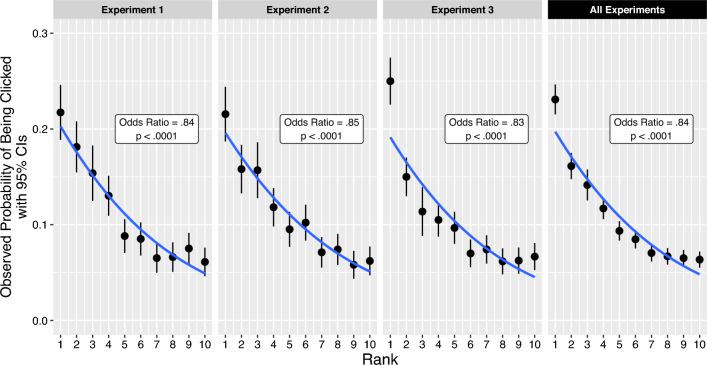
Higher rank (X-axis) is associated with an increase in an accurate result's click likelihood (Y-axis). This relationship (blue line) is stable across experiments (columns). The blue lines are fitted by the logistic regression model and show the predicted probabilities of each rank being clicked, while the black points show the observed probabilities. For example, the left-most point in the left-hand panel (“Experiment 1”) shows that an accurate result appearing at the highest rank in this experiment had a 22% likelihood to be clicked. Each datapoint represents probabilities generated from 598 to 3196 individuals' click decisions, depending on the experiment and rank displayed (since not all participants were exposed to accurate information in the first three ranks). Error bars represent 95% Confidence Intervals of the sample proportions.

Across all experiments, our data supported H0: accurate results in higher ranks were more likely to be clicked. Figure 6 provides a detailed overview of the rank-click relationships for each experiment in isolation and with their data combined (represented in each panel). For every drop in position by 1, the odds of an accurate result being clicked decreased by 16% in Experiment 1 (OR = 0.84, 95% CI [0.82, 0.86]), 15% in Experiment 2 (OR = 0.85, 95% CI [0.83, 0.87]), 17% in Experiment 3 (OR = 0.83, 95% CI [0.82, 0.85]), and 16% across all experiments (OR = 0.84, 95% CI [0.83, 0.85]; all *p*s < 0.0001). This effect was robust when our models controlled for the presence and rank of misinformation on the page and the specific search engine the results were attributed to in Experiments 1 and 2 (although the strength of this relationship differed under each warning banner: see the Warnings subsection).

We also found support for H1, in that participants trusted accurate results they clicked more than accurate results they did not click by 0.71 scale points in Experiment 1 (*F*(1, 979) = 245.7, *p* < 0.0001), 0.59 scale points in Experiment 2 (*F*(1, 995) = 68.62, *p* < 0.0001), 0.61 scale points in Experiment 3 (*F*(1, 1191) = 95.71, *p* < 0.0001), and 0.69 scale points in the aggregate data (*F*(1, 4008) = 357.45, *p* < 0.001). This effect was stable when the models controlled for the result's rank, its accuracy, the presence and rank of misinformation on the page, and the specific search engine the results were attributed to in Experiment 1.

**Figure 7:**
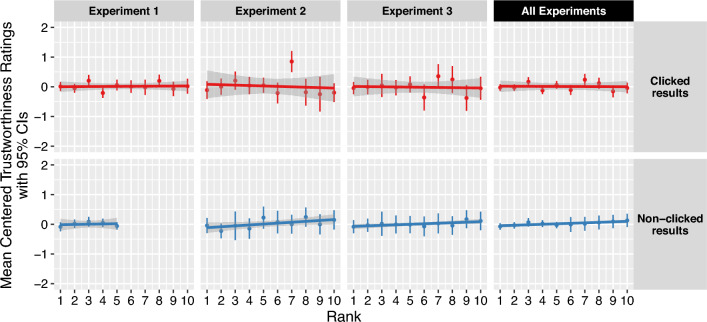
Rank (X-axis) does not affect the evaluation of trustworthiness (Y-axis, mean-centered) of accurate results. This lack of relationship is robust across experiments (columns) and for clicked results (top row, red) as well as non-clicked results (bottom row, blue). The trend lines represent the predicted change in trustworthiness ratings per unit decrease in rank fitted by the linear regression models. Each dot represents 19 to 431 trustworthiness evaluations, depending on the rank, experiment, and click category shown. For instance, the third point from the left in Experiment 1’s top panel shows that participants who clicked an accurate result in the third rank on the page gave it trustworthiness scores that were about 0.25 scale points higher than the average trustworthiness rating that participants gave the results they clicked across all ranks in this experiment. All slopes for rank had *p*s > 0.05. The figure separates ranking for clicked (top) and non-clicked (bottom) to illustrate that the non-effect is robust for both types of results. Error ranges represent 95% confidence intervals of the centered trust ratings within the rank and click category for each experiment.

Given the effect of rank on click probability, and the relationship between clicking and trustworthiness evaluations, it seemed plausible that rank could impact the perceived trustworthiness of results on search pages (H2). However, that is not what we found. Unlike click behavior, evaluations of trustworthiness did *not* vary with the rank of accurate search results in linear regressions (all *p*s > 0.05), leaving H2 unsupported. Figure 7 demonstrates this null effect, which was robust for both clicked and non-clicked results, as well as when our models controlled for the presence and rank of misinformation, the presence and type of warning banner in Experiment 2, and the specific search engine the results were attributed in Experiment 1.

### Responses to misinformation

Responses to misinformation in high-ranked search results were nearly identical across the experiments: While participants recognized the misinformation (as seen through low clicks and trust ratings), the presence of misinformation did not impact how much people clicked or trusted accurate information below it.

First, misinformation was rarely clicked and highly distrusted. Across all experiments, only 2.6% of participants who were exposed to inaccurate results clicked on these results. Misinformation results were clicked at a significantly lower rate than accurate information displayed in the same position, as seen via Chi-square tests (results from aggregate data: rank 1: *X*^2^(1, 1996) = 68.47, *p* < 0.001; rank 2: *X*^2^(1, 1996) = 46.16, *p* < 0.001; rank 3: *X*^2^(1, 3196) = 153.96, *p* < 0.001. The tests for each individual experiment aligned with these results and can be found in the Supplementary Information). As expected in light of this finding and the trust-click association, participants also trusted misinformation results less than accurate results, by 1.70 scale points in Experiment 1 (*F*(1, 781) = 1224.4), 0.91 points in Experiment 2 (*F*(1, 995) = 111.12), 0.73 points in Experiment 3 (*F*(1, 1191) = 54.33), and 1.56 points across all experiments (*F*(1, 5190) = 1696.6, all *p*s < 0.0001). Thus, both H3a and H3b were supported and suggest that the misinformation manipulation was salient.

Despite the clear responses to misinformation itself, the presence of misinformation in a search result did not affect participants’ propensity to click accurate information located immediately below it (found in nonsignificant Chi-square tests measuring clicks on accurate results below misinformation between conditions; all *p*s > 0.05). H4a is thus unsupported.

**Figure 8:**
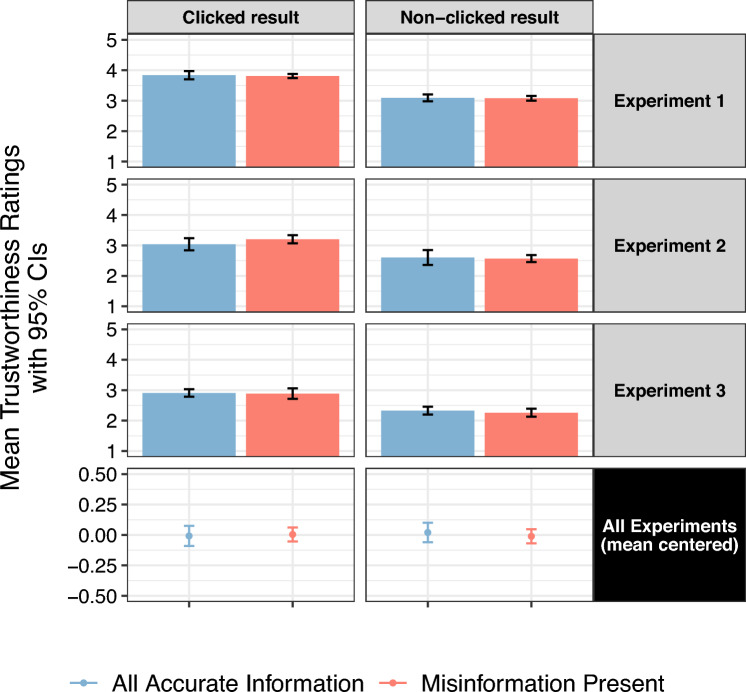
The presence of misinformation (red bars and points) had no effect on trust (Y-axis) in all of the surrounding accurate results on the page compared to when none of the results on the page contained misinformation (blue bars and points). This null effect was stable across experiments (rows) and for both clicked (left column) and non-clicked (right column) results. The Y-axis displays the mean trustworthiness ratings given to accurate results across all ranks on the page by participants who saw a misinformation result on the page (the red bars and points on the right side of each panel) compared to those who saw no misinformation result anywhere on the page (the blue bars and points on the left). Each bar in the figure represents trustworthiness ratings from 96 to 1473 participants, depending on the experiment and click category displayed. For example, the red bar in the right-hand panel in the “Experiment 1” row shows that when participants evaluated an accurate result they had *not* clicked after seeing misinformation in another result, they gave it an average trustworthiness rating of about 3.1 scale points, and the average for these results when there was no misinformation present (blue bar) was similar at about 3.1 scale points. For the data from all experiments (bottom row), ratings are mean centered within each experiment and the respective click category because the experiments’ overall mean trustworthiness ratings differed for both clicked and non-clicked results, so we needed to standardize them within each experiment before aggregating them. Error ranges represent 95% Confidence Intervals of the means.

The presence of misinformation also did not cause people to distrust the accurate information they had seen, leaving H4b unsupported. Figure 8 illustrates that there was no significant difference in how trustworthy people perceived accurate results when a search page showed misinformation in a high rank (all *p*s > 0.05). Some literature predicts that misinformation exposure will most heavily affect people’s perceptions of accurate content that comes right afterward, but people may also not look past the misinformation result, leaving only their trust in accurate information above it to be influenced. To thoroughly investigate these possibilities, we reran these models on trust ratings given to the accurate results that came (1) immediately below misinformation; and (2) above the misinformation, both compared to the same-ranked accurate results in the conditions without misinformation. Again, the models run on both of these subsets of accurate results showed that misinformation did not cause people to distrust these specific results (all *p*s > 0.05; see the Supplementary Materials, Sect. 8 for full model outputs). This non-effect was robust for clicked and non-clicked accurate results, as well as when our models controlled for the presence and rank of misinformation, the presence and type of warning banner in Experiment 3, and the specific search engine the results were attributed to in Experiment 1 and Experiment 2.

### Responses to warnings

Experiment 3 introduced the additional treatment of an information reliability warning banner at the top of the result page. We performed a between-subject test of two different warnings (shown in “Methods”, Fig. 5): the “evolving results” warning used by Google that says “the results are changing quickly” and a version we created called the “source reputation” warning. (A control condition showed no warning banner.)

**Figure 9:**
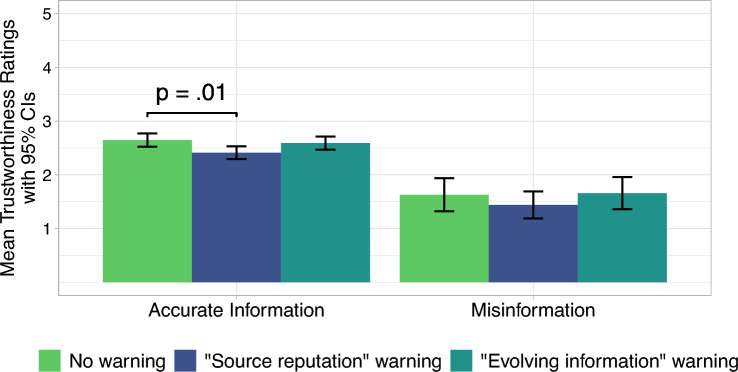
The “source reputation” warning decreases trust in accurate information by about a quarter of a scale point, thus representing the opposite of the expected outcome. The bars show mean trustworthiness ratings (Y-axis) for accurate and misinformation results depending on the warning condition: no warning (light green bars on the left), the “source reputation” warning (dark green bars in the middle), or the “evolving information” warning (dark blue bars on the right). Each bar in the figure represents 50 to 352 trustworthiness ratings depending on the type of result and the warning condition. Error ranges represent 95% Confidence Intervals of the means.

We found that the “source reputation” warning decreased people’s trust in accurate results, while Google's “evolving results” warning had no impact (*F*(2, 1195) = 4.68, partial eta squared = 0.006, *p* = 0.013). Figure 9 reports the effect of warnings on mean trustworthiness ratings of results, for accurate results (on the left) and misinformation results (on the right). Participants rated accurate results lower in trustworthiness by 0.245 points when they saw the “source reputation” warning (estimated marginal mean from model = 2.47, 95% CI from model [2.35, 2.59]), compared to those who saw no warning (estimated marginal mean = 2.72, 95% CI from model [2.60, 2.84]). This effect was robust when our model controlled for whether misinformation was present on the page, whether participants were rating results they had clicked or not, and the rank of the result being rated (which had no effect on trust, as seen previously). In contrast, the “evolving information” warning had no effect on trust levels compared to the control. The right side of the figure shows that neither warning had a significant effect on trust levels in misinformation, though a post-hoc power analysis showed that we did not have enough ratings of the misinformation results to detect a significant decrease in trust (this lack of impact could also be a floor effect, since although the perceived trustworthiness of the accurate results was fairly low, the perceived trustworthiness of the misinformation was near the lowest possible measurement point at the baseline; see “Discussion” and Sect. 10 of the Supplementary Information for details on the power analysis and limitations). Given that one of the warning banners decreased trust in the accurate information, our data partially support H5 and suggest that these types of broad warning banners can backfire by unintentionally increasing distrust in accurate information.

This finding raises two concerns. First, the decreased trust in accurate information caused by the “source reputation” warning could theoretically steer people toward trusting and clicking misinformation instead. Second, decreased trust in accurate information could cause people to rely on other unreliable cues in the search environment (such as rank) more heavily when making their trustworthiness evaluations and click decisions, since Cognitive Miser Theory predicts that people are especially likely to rely on heuristics in their environment to guide their decisions when the environment is unfamiliar or otherwise violates the expectation^17^.

Our data do not support these concerns, however. Across warning conditions, misinformation’s trust ratings and click rates remained equally low, and misinformation still had no spillover effects on clicks or trust in accurate information below it. Moreover, neither warning changed the (null) effect of rank on trust. Finally, participants who saw the “source reputation” warning were actually *less* likely to click on high-ranked accurate results compared to participants who saw the other warning, though neither warning condition showed significant differences in this relationship relative to the control.

**Figure 10:**
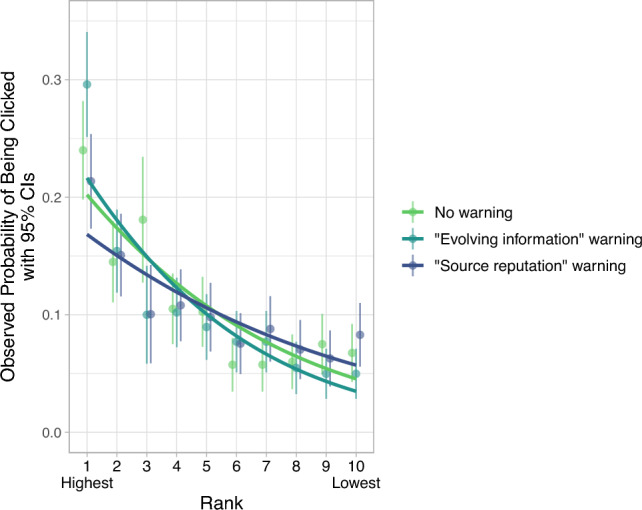
Misinformation warning banners may moderate people’s reliance on rank when deciding which results to click. The “source reputation” warning (dark blue line) weakened the effect of rank (X-axis) on participants’ likelihood of clicking an accurate result (Y-axis) compared to when participants saw identical search results under the “evolving information” warning banner (dark green line). Each line represents click probabilities estimated by the logistic regression model, while each point represents the observed probabilities. For instance, the dark green point at rank 1 shows that the highest-ranked result was clicked by about 30% of participants in the “evolving information” warning group. Each datapoint represents probabilities generated from 199 to 402 individuals' click decisions, depending on the warning and misinformation-presence conditions. Error bars represent 95% Confidence Intervals of the proportions of participants who clicked the accurate result at each rank.

Figure 10 illustrates the relationship of rank and clicking in response to the warnings. The logistic regression model showed a significant interaction between warning condition and rank for click likelihood (*p* < 0.01), which we unpacked using post-hoc pairwise comparisons of the rank slopes between each warning condition. The most moderate slope in the figure is that of the “source reputation” warning (darkest/blue line), which was associated with a 14% decrease in odds of clicking per one-step drop in rank (N = 398, OR = 0.86, 95% CI [0.82, 0.91], *p* < 0.001), whereas the “evolving information” warning was associated with a significantly larger 21% decrease (N = 402, OR = 0.79, 95% CI [0.74, 0.84], *p* < 0.001; pairwise contrast *p* < 0.01). While the differences between these two slopes was significant, neither of these estimates significantly differed from the “no warning” group's 17% decrease (N = 400, OR = 0.84, *p* < 0.0001).

## Discussion

Across three large online experiments, which directly measured the perceived trustworthiness of individual search results while randomizing their order in a ranked list, neither rank nor the presence of misinformation affected the extent to which people trusted information in search results’ information and sources. This null effect remained when we changed the specific search engine and added elements of uncertainty to the search setting (e.g. misinformation results, queries lacking consensus, unfamiliar sources for the search results, and warnings about unreliable information).

The finding that the rank does not impact the perceived trustworthiness of search results has practical and theoretical implications. On the practical side, there may be less need to worry about search engines diminishing people’s trust toward authoritative sources (such as the CDC in the case of Covid-19) if their information is not listed near the top of the page. The absence of an association between rank and perceived trustworthiness revealed in our experiments suggests that reliance on rank for clicking may instead be due to other factors. One possibility we considered is that people click on high-ranked results because of a widespread belief that highly-ranked results are likely to be most *relevant* to the search query^14^. However, we ran models of rank’s impact on relevance ratings of the results and found no significant relationships in any of the experiments (see Supplementary Information Sect. 9). Thus, other Cognitive Miser motivations are more likely to explain the rank-click relationship we observed in our studies, such as the mere convenience of being able to find information quickly.

Our findings also add to the literature on the potential effects of misinformation and approaches to address it in high-stakes information-seeking contexts. First, some potentially good news: contrary to the suggestions of past work^2,27,28^, misinformation in our study had no negative effects on people’s propensity to click or trust accurate results beneath it. We note that the observed immunity to misinformation could have been skewed by our samples' demographics (see below).

At the same time, our study is among the first to show that the warning-banner approach to combat misinformation has mixed effects when people are evaluating the trustworthiness of search results. Although a warning about unreliable sources showed some potential to reduce people’s trust in results with misinformation, this warning significantly reduced their trust in results with *accurate* information. This decreased trust in accurate information represents a backfire effect: while it might be a rational outcome in the sense that the warning mentions “sources” plural, it is certainly not the outcome intended by the search engines who develop these warnings. This outcome also matches findings from recent studies showing that general misinformation approaches can backfire by casting doubt on genuinely valid information^27,35,36^. That said, our analysis of the rank-click relationship under different warning conditions suggests a more nuanced effect of this warning: People exposed to the “source reputation” warning showed a decreased likelihood of clicking on high-ranked results compared to people exposed to the “evolving information” warning. That is, instead of relying more on rank as a heuristic to guide click decisions when the warning made the uncertainty salient, people became more cautious and relied on rank less. Thus, the “source reputation” warning may have served as a double-edged sword, giving rise to healthy skepticism that made people consider more of the information on the search page. In sum, the effects of nonspecific warnings are multifaceted and warrant more research, particularly in search engines.

Despite the apparent robustness of our results, as evidenced by their consistency across searches with varying levels of uncertainty and the replication of past studies’ rank-click relationship, our studies are limited in the following ways.

First, research has shown that susceptibility to misinformation may be concentrated among a small group that we did not specifically target in our recruitment^48,49^. Our participants were recruited through Prolific Academic, rendering them younger, more politically liberal, and more supportive of vaccination than the overall population. The non-representativeness of online samples is a common problem in modern-day survey research^50^. Future work might profit by studying the effects of misinformation in online search and mitigation approaches on those most susceptible to it.

In addition, we lacked the requisite number of people who rated their trust in the misinformation in Experiment 3 to be able to detect if the “source reputation” warning had a similar ability to decrease people’s trust in it. Descriptively, there was a similar decrease in trust in this condition. Regardless of whether this warning impacts trust in misinformation, our finding that the warning decreased trust in accurate information could be cause for concern, since it suggests that this warning leads to indiscriminate distrust of all information presented by search engines.

Second, our experimental setup precludes testing the relationship between perceived trustworthiness and click probability (H1) in the presumed causal direction (i.e., perceived trustworthiness causes clicking). While one can argue that participants may give higher trustworthiness ratings to the result they clicked in the previous step as a form of rationalization, a la Self Perception Theory^51^, we attempted to avert this possibility by not explicitly reminding participants when the result they were assigned to rate was the one they had clicked. Rationalization could also occur with a reversed design, in which subjects first rate the trustworthiness of every result on a page, then click one. In such a design, participants could feel more pressure to click on the result to which they had assigned the highest trustworthiness.

Third, with crisis events, the veracity of information is often uncertain, which sometimes clouds the distinction between accurate information and misinformation. For our study, we used blatantly false claims about Covid-19, which were both unsupported by peer-reviewed scientific reports and rated as inaccurate by participants in pretests. We have shown that blatant misinformation fails to undermine confidence in accurate search results that follow, but further research might reveal whether these effects extend to more subtle forms of misinformation that may appear in search results.

Our experiments were focused on health information, and more specifically Covid-19, but it is possible that results for Covid-19 search queries do not generalize to topics other than health. Health-related searches may require greater deference to professional expertise compared to searches about lifestyle topics, sports, or shopping, for example. According to Palen et al.^21^, “when considering emergencies, stakes are often quite high, so credibility must be established quickly, although often only partially, before one decides what to do (or not to do) with [the information]” (p. 13). Indeed, search in important contexts like health and crisis response is where it is most important to understand, like we do here, the trustworthiness evaluations and the factors that impact them, for example the presence of misinformation. Future work should explore whether our findings hold for other types of searches.

Finally, despite their greater internal validity, lab experiments’ external validity is not as robust as that of field experiments. We do have some evidence that our treatments worked (e.g. the lower rate of clicks on misinformation, and the decrease in average trustworthiness ratings as we added uncertainty in the forms of unfamiliar sources starting in Experiment 2 and uncertain queries in Experiment 3). Nevertheless, we believe our results are informative and extend the current literature on how search engines influence people’s trust in a society increasingly reliant on computer searches for critical information.

### Supplementary Information

Supplementary Information.

## Data Availability

The preregistrations, code, anonymized datasets, and detailed results for each analysis are available at the anonymous OSF repository for this project (https://osf.io/n2b3s/?view_only=23f4248f7156474885376a26e402d745).
