# Supplementary material for: Misinformation does not reduce trust in accurate search results, but warning banners may backfire
Source: Sci Rep. 2024 May 14;14:10977. doi: 10.1038/s41598-024-61645-8 (PMC11094033; doi:10.1038/s41598-024-61645-8)
Supplement: Supplementary file 1 — Supplementary Information. [file 41598_2024_61645_MOESM1_ESM.pdf]

Supplementary Information for

# Misinformation doesn't reduce trust in accurate search results, but warning banners may backfire

Sterling Williams-Ceci[1,3]*, Michael W. Macy[1,2], Mor Naaman[1,3]

[1] Department of Information Science, Cornell University, Ithaca, NY, USA
[2] Department of Sociology, Cornell University, Ithaca, NY, USA
[3] Cornell Tech, New York, NY, USA

*Corresponding Author: Sterling Williams-Ceci (scw222@cornell.edu)

**Table of Contents:**

1. Pretests: misinformation, unfamiliar sources, uncertain queries
2. Materials: queries, search results & sources
3. Survey procedure
4. A priori power analysis
5. Pilot study
6. Descriptive statistics
7. Trustworthiness ratings
8. Reported and pre-registered analyses
    a. Effect of rank on click likelihood for accurate results
    b. Effect of misinformation on click likelihood of results in a given rank
    c. Effect of misinformation on click likelihood for accurate results below it
    d. Associations of clicking, information quality, and warnings with trust
    e. Effect of rank on trust
    f. Effect of misinformation on trust in accurate results
9. Exploratory analyses
10. Post-hoc power analysis for warnings' effects

## 1. Pretests

Below, we detail the validation pretest procedures and results for each experiment. All participants recruited in the pretests were excluded from participating in any of the three main experiments.

**Misinformation for Experiments 1 & 2.** Our experiments involved manipulating whether one of the top three search results had misinformation; however, people often disagree over what information constitutes "misinformation," especially in rapidly-emerging situations such as Covid-19. To ensure that our manipulation was effective, we pretested five examples of misinformation results for each Covid-19 query. We recruited 30 participants from different geographic areas and socioeconomic backgrounds. As in the main experiment, we showed them pages of ten results for each query in a randomized order. Five of these results were examples that had misinformation, such as claiming that the Covid-19 vaccines cause cancer. The other five results had what we defined as "accurate information," which were results from reputable medical sources that said the vaccines are safe. We asked participants to click on any results that they believed had false information. We used the three misinformation results that were selected most frequently for each query among those chosen by at least 80% of participants, and used these in our main experiment. (Note: two of the misinformation results had a tie for one of the queries, but both came from the same source; to diversify the sources of misinformation, we used the fourth most frequently-picked result in place of one of the tied ones). This pretest also confirmed that our accurate results were rarely chosen by participants as being false. See the **Materials** section for the final search results we used in the main experiment.

**Uncertain Queries for Experiment 3.** Experiment 3 used three new Covid-19 queries whose answers were highly uncertain and unestablished among the medical community. We searched Google to find several examples of questions about Covid-19 that had a lack of consensus among medical sources (defined either as queries with medical sources saying that the answers were still unknown, or queries with multiple medical sources providing answers that were in opposition to each other). We then pretested these queries on a sample of participants from Prolific (n = 80) to verify that participants would be aware of these queries' uncertainty. We showed the queries in randomized order and asked these participants to indicate their answers (options were "yes," "no," or "I don't know"). We also mixed in some questions that were more certain to give participants some questions they would feel confident of the answers to; we did this to prevent them from changing their answers to the uncertain questions in order to appear more knowledgeable. In addition, we asked participants to rate how personally relevant the questions were to them (measured with a 5-point Likert scale anchored at "extremely irrelevant" and "extremely relevant"), so that we could ensure the questions we used would still be interesting to participants during the main experiment.

We computed two uncertainty metrics for each query: the percentage of participants who selected "unsure," and an ordinal value for how evenly split the answers were among the three options given (e.g., a query that had exactly 33% of respondents answering each option would have a value of 1). We compared the top queries for each of these metrics and found that the ones that had the highest percentage of "I don't know" responses were generally lower in relevance ratings than the queries that were the highest in dispersion, so we chose the top three queries with

the highest dispersion values. The final selected queries used in Experiment 3 are shown in the **Materials**.

**Misinformation for Experiment 3.** Due to the lack of surefire answers to the new, uncertain queries used in Experiment 3, we created our own examples of misinformation results (containing generalized misinformation about the pandemic). We doctored five pieces of misinformation to look like the content of real search results, and we pretested them on a new group of Prolific participants (n = 80). Similar to our pretest of the queries, we showed participants these search results' misinformation mixed in randomized order with search results containing accurate information. We asked participants to rate the perceived accuracy of the content (measured using a 6-point Likert scale anchored at "definitely false" and "definitely true"), and the perceived likelihood of seeing it on a page of search results (measured using a 7-point Likert scale anchored at "very unlikely" and "very likely"). We added this second question to find examples that people recognized as false but also felt could plausibly appear in online searches, in order to make our experimental setup more realistic.

For each query, there were significant differences in average accuracy and realism ratings of the results with accurate information and misinformation, confirming that our examples were perceived to be misinformation. We then conducted pairwise t-tests of accuracy and realism ratings among the individual misinformation results for each query (with Bonferroni p value adjustments). When there was a result excluding those that had significantly higher accuracy ratings than the others, we excluded it from consideration. (There were never any significant differences in realism ratings among these results). From the remaining results, we selected the three with the lowest median accuracy scores for each query. The final misinformation results had an overall average accuracy rating of 1.5 out of 6 scale points, and an overall average realism rating of 3.1 out of 7 scale points.

**Unfamiliar sources for Experiments 2 & 3.** To compare whether people showed different search tendencies from Experiment 1 when they did not have prior knowledge about the search results' sources, we obtained several source URLs from real Google search results that were uncommon (appearing on several pages after the first page of results and unrecognizable to the researchers). To validate our judgments and find the sources that were most unknown to participants on Prolific, we ran a pretest of these sources on a separate group of participants (n = 66) from those recruited in our main experiments. We showed these participants a series of URLs in randomized order, including: 18 uncommon websites, 8 common websites, and 15 fake websites whose names we fabricated (our goal was to use real websites that were unknown, but we included the fake sites in case all of the real sites that we believed were unknown were actually widely recognized by participants. This scenario did not materialize, so we were able to discard the fake sites from consideration). For each, we asked participants to indicate whether they recognized the website (options were "Yes, I recognize this website" "No, I do not recognize this website," and "I'm not sure if I recognize this website"). We also asked participants to indicate whether they believed the website sounded real or fake (options were "This website sounds real," "This website sounds fake," or "I'm not sure if this website sounds real or fake"); we included this question to eliminate websites that were frequently rated as sounding fake, to support the realistic setup of our main experiments.

We started by summing the percentages of participants who answered that they did not recognize a site or were unsure if they recognized it (hereafter referred to as “unknownness”). This led us to discard an outlier that had the lowest unknownness score. We then consulted the answers to the question about whether each site sounded real or fake, and eliminated four more sites that had low percentages of participants who indicated that they sounded realistic. We were then left with 13 unknown sites, all of which had unknownness scores of at least 87.5%

## 2. Materials

**Queries.** Participants in Experiments 1 and 2 were randomly assigned to complete the survey for one of the following queries, shown in either a Google or DuckDuckGo search bar depending on the condition assignment:

1. “Are covid vaccines safe for children?”
2. “Can covid vaccines cause infertility later in life for children?”
3. “Can covid vaccines cause cancer in children?”

In Experiment 3, participants were randomly assigned to complete the survey for one of the following new queries that were pretested and found to be highly uncertain to participants (see **Pretests** for details):

1. “Can animals transmit covid-19 to humans?”
2. “Is it recommended to wear two face masks to prevent covid-19 infection?”
3. “Is natural immunity from covid-19 more effective than immunity from the vaccine?”

**Search results.** We used Google to find real search results with accurate information and misinformation for the queries used in each experiment. The only exception to this was in Experiment 3, whose queries lacked clear misinformation on Google at the time of the experiment: as a result, we created our own examples of misinformation for each of these queries. In two separate pretests, we validated our misinformation results by asking participants to select results they believed to be misinformation from a list with a mixed list. One of these was randomly selected to be shown to each participant in the misinformation conditions at the corresponding rank, and was surrounded by the first nine of the accurate results in a randomized order. Experiment 1 showed the results as they originally appeared on Google with their actual source URLs; we stripped these URLs from each result in Experiment 2 and instead assigned each text snippet to be paired with unfamiliar sources (see below). Experiment 3 used a new set of results for the new queries, with the same source-matching procedure as in Experiment 2. See Figure S1 - Figure S14 below for the exact results used.

The following are the search results as shown in Experiment 1 for each query; the same results were used in Experiment 2 with different sources:

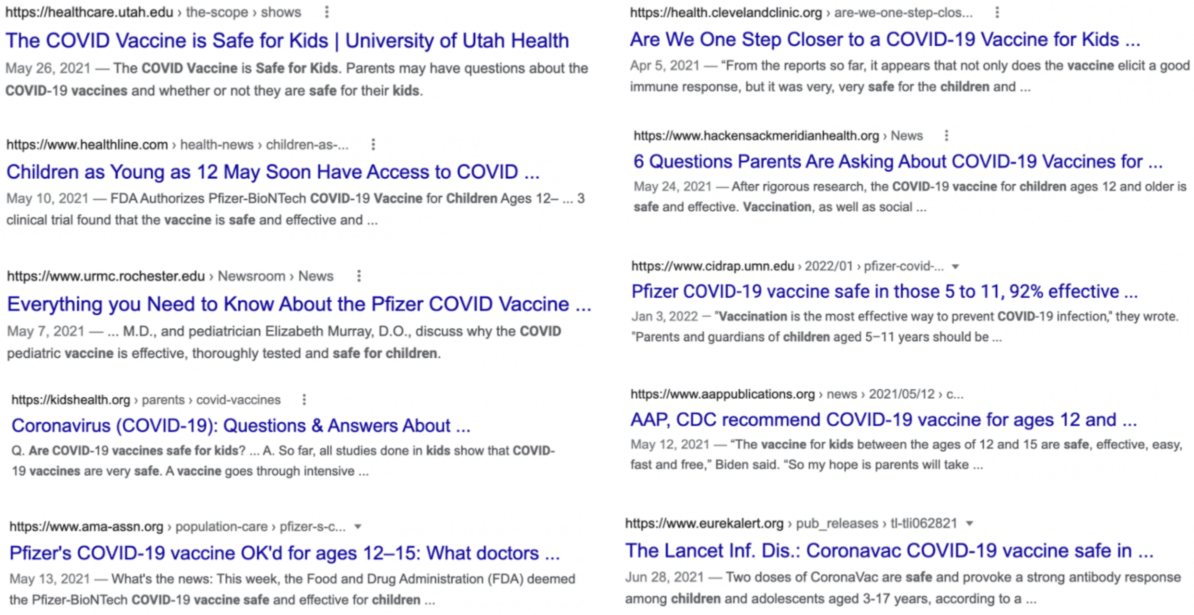

https://healthcare.utah.edu › the-scope › shows
The COVID Vaccine is Safe for Kids | University of Utah Health
May 26, 2021 — The **COVID Vaccine** is **Safe for Kids**. Parents may have questions about the **COVID**-19 **vaccines** and whether or not they are **safe** for their **kids**.

https://www.healthline.com › health-news › children-as-...
Children as Young as 12 May Soon Have Access to COVID ...
May 10, 2021 — FDA Authorizes Pfizer-BioNTech **COVID**-19 **Vaccine** for **Children** Ages 12– ... 3 clinical trial found that the **vaccine** is **safe** and effective and ...

https://www.urmc.rochester.edu › Newsroom › News
Everything you Need to Know About the Pfizer COVID Vaccine ...
May 7, 2021 — ... M.D., and pediatrician Elizabeth Murray, D.O., discuss why the **COVID** pediatric **vaccine** is effective, thoroughly tested and **safe for children**.

https://kidshealth.org › parents › covid-vaccines
Coronavirus (COVID-19): Questions & Answers About ...
Q. **Are COVID**-19 **vaccines safe for kids**? ... A. So far, all studies done in **kids** show that **COVID**-19 **vaccines** are very **safe**. A **vaccine** goes through intensive ...

https://www.ama-assn.org › population-care › pfizer-s-c...
Pfizer's COVID-19 vaccine OK'd for ages 12–15: What doctors ...
May 13, 2021 — What's the news: This week, the Food and Drug Administration (FDA) deemed the Pfizer-BioNTech **COVID**-19 **vaccine safe** and effective for **children** ...

https://health.clevelandclinic.org › are-we-one-step-clos...
Are We One Step Closer to a COVID-19 Vaccine for Kids ...
Apr 5, 2021 — "From the reports so far, it appears that not only does the **vaccine** elicit a good immune response, but it was very, very **safe** for the **children** and ...

https://www.hackensackmeridianhealth.org › News
6 Questions Parents Are Asking About COVID-19 Vaccines for ...
May 24, 2021 — After rigorous research, the **COVID**-19 **vaccine** for **children** ages 12 and older is **safe** and effective. **Vaccination**, as well as social ...

https://www.cidrap.umn.edu › 2022/01 › pfizer-covid-...
Pfizer COVID-19 vaccine safe in those 5 to 11, 92% effective ...
Jan 3, 2022 — "**Vaccination** is the most effective way to prevent **COVID**-19 infection," they wrote. "Parents and guardians of **children** aged 5–11 years should be ...

https://www.aappublications.org › news › 2021/05/12 › c...
AAP, CDC recommend COVID-19 vaccine for ages 12 and ...
May 12, 2021 — "The **vaccine** for **kids** between the ages of 12 and 15 are **safe**, effective, easy, fast and free," Biden said. "So my hope is parents will take ...

https://www.eurekalert.org › pub_releases › tl-tli062821
The Lancet Inf. Dis.: Coronavac COVID-19 vaccine safe in ...
Jun 28, 2021 — Two doses of CoronaVac are **safe** and provoke a strong antibody response among **children** and adolescents aged 3-17 years, according to a ...

Figure S1: Accurate results for the query "Are covid vaccines safe for children?" in Experiment 1 and Experiment 2.

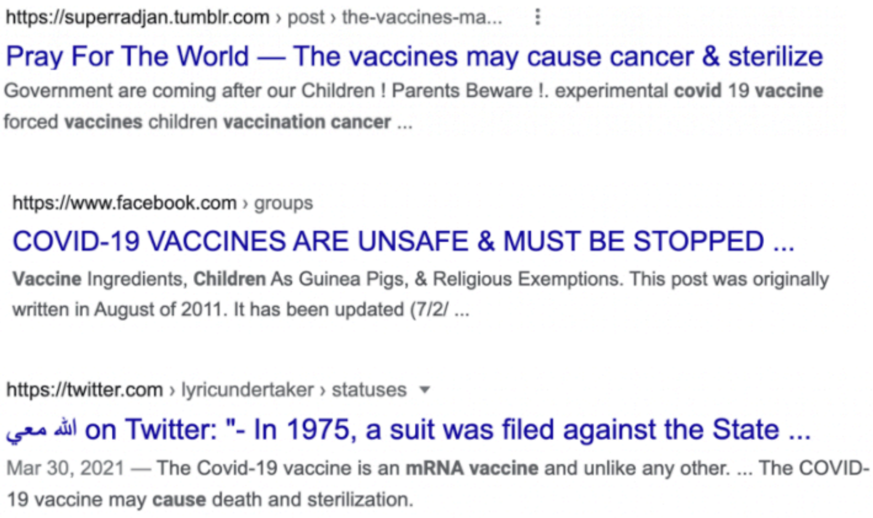

https://superradjan.tumblr.com › post › the-vaccines-ma...
Pray For The World — The vaccines may cause cancer & sterilize
Government are coming after our Children ! Parents Beware !. experimental **covid** 19 **vaccine** forced **vaccines** children **vaccination cancer** ...

https://www.facebook.com › groups
COVID-19 VACCINES ARE UNSAFE & MUST BE STOPPED ...
**Vaccine** Ingredients, **Children** As Guinea Pigs, & Religious Exemptions. This post was originally written in August of 2011. It has been updated (7/2/ ...

https://twitter.com › lyricundertaker › statuses
الله معي on Twitter: "- In 1975, a suit was filed against the State ...
Mar 30, 2021 — The Covid-19 vaccine is an **mRNA vaccine** and unlike any other. ... The COVID-19 vaccine may **cause** death and sterilization.

Figure S2: Misinformation results for the query "Are covid vaccines safe for children?" in Experiment 1 and Experiment 2.

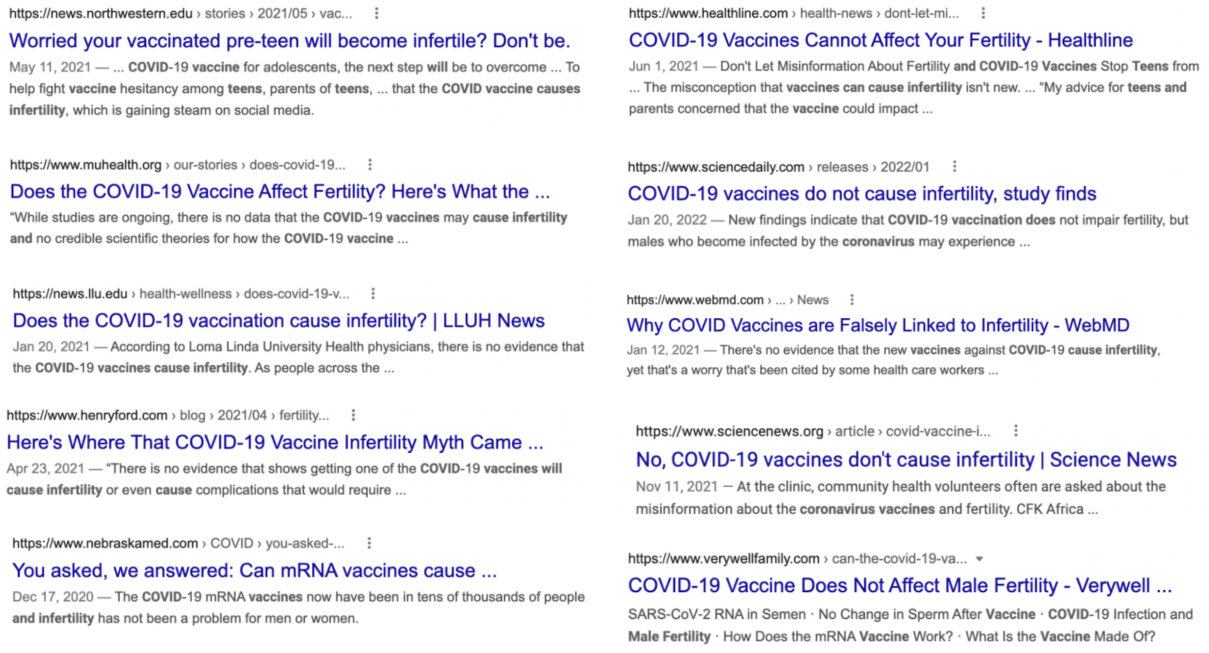


Figure S3: Accurate results for the query "Can covid vaccines cause infertility later in life for children?" in Experiment 1 and Experiment 2.

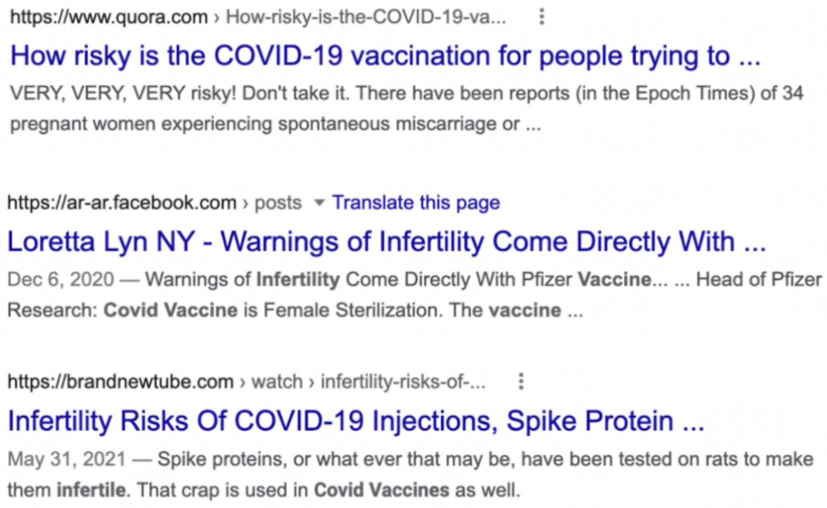

Figure S4: Misinformation results for the query "Can covid vaccines cause infertility later in life for children?" in Experiment 1 and Experiment 2.

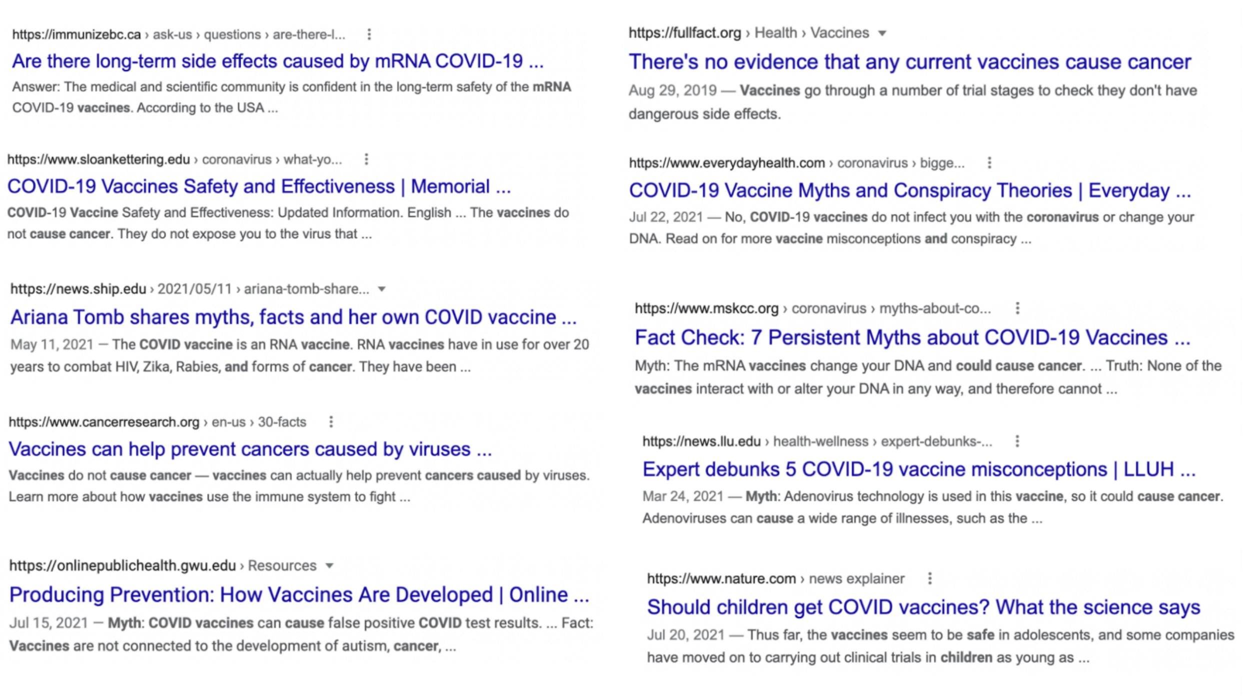


Figure S5: Accurate results for the query "Can covid vaccines cause cancer in children?" in Experiment 1 and Experiment 2.

https://x.facebook.com › CDC › photos

**CDC - If your child is Medicaid eligible, American Indian or ...**

Jun 8, 2021 — Don't do it. Research. **mRNA** is in all child **vaccines** now and (HIV aborted fetal cells, since 1987) ... 2 H351 Suspected of **causing cancer**.

https://twitter.com › status Translate this page

**Prasaanth Saran on Twitter: "The mRNA vaccine is coming to ...**

Jan 9, 2021 — The **mRNA vaccine** is coming to the human race for the first time. This Genetic modification that **causes** the body to **trigger cancer**.

https://www.reddit.com › comments › what_are_some_...

**What are some strange things or symptoms your patients have ...**

For example, a recent patient blamed her jaundice on the **covid vaccine** she received 3 days ... Sounds to me like the **COVID vaccine caused** pancreatic **cancer**.

Figure S6: Misinformation results for the query "Can covid vaccines cause cancer in children?" in Experiment 1 and Experiment 2.

The following are Experiment 3's search results for each query (these were randomly paired with the unfamiliar sources used in Experiment 2):

**Pets don't transmit COVID-19**

**Dogs** and **cats** in contact with infected **humans** have tested positive for **COVID-19**. However, there are no evidence to date that they **can transmit COVID-19 to humans**. Learn More.

**People probably caught coronavirus from minks. That's a ...**

After reports of infected dogs emerged from Hong Kong, they said there was no evidence **animals could transmit** the virus to **humans**.

**No evidence that animals can transmit the coronavirus to ...**

No evidence that **animals can transmit** the coronavirus to **humans** · There is no current evidence of dogs playing a role in the **spread** of **COVID-19**.

**Animals unlikely to spread Covid-19 to humans, but ...**

There's no evidence that **animals** are playing a significant role in the **spread** of **coronavirus** to **humans**, but precautions **can** help keep people ...

**Pets are Blameless in Spread of COVID-19 Coronavirus**

This is a case of **human-to-animal** transmission and means that dogs **can carry** low levels of the virus but don't become sick.

**Dog coronavirus jumps to humans, with a protein shift**

... provide clues to how they **transmit** to **humans** from **animals**. A new canine **coronavirus** was first identified in two Malaysian **human** patients ...

**Can pets transmit coronavirus to humans? No evidence so far ...**

Experts say canine coronavirus is different from **COVID-19**, which is spreading now. And unlike **COVID-19**, the canine coronavirus has a vaccine ...

**Researchers identify first case of animal-to-human COVID ...**

At **first**, **researchers** thought white-tailed deer were being infected by **people**, but new evidence shows it may also work the other way around.

**A deer may have passed COVID-19 to a person, study suggests**

She hopes "this **will** be the future of biosurveillance, where we **will** have to look across the spectrum of the **animal** kingdom—not just **humans** in ...

**Your pets can catch coronavirus, but don't spread it**

Cats **and** dogs **don't** tend to **get** very sick or transmit **COVID**-19, scientists say.

Figure S7: Accurate results for the query "Can animals transmit covid-19 to humans?" in Experiment 3.

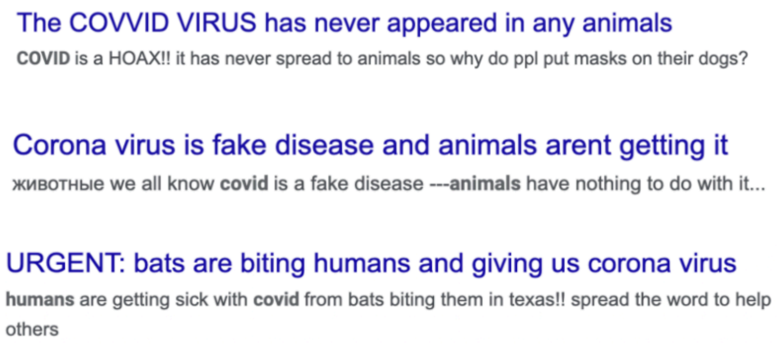


Figure S8: Misinformation results for the query "Can animals transmit covid-19 to humans?" in Experiment 3.

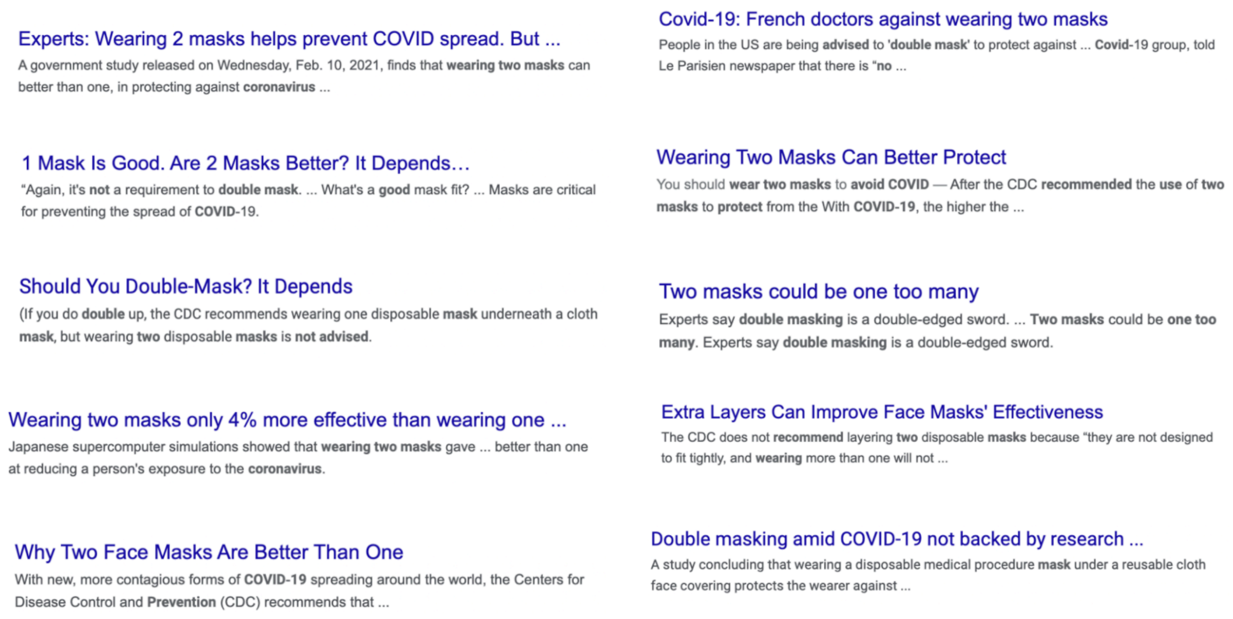


Figure S9: Accurate results for the query "Is it recommended to wear two face masks to prevent covid-19 infection?" in Experiment 3.

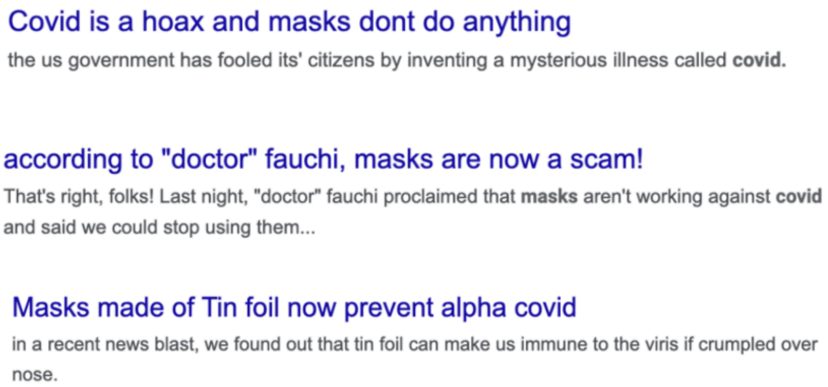

Covid is a hoax and masks dont do anything
the us government has fooled its' citizens by inventing a mysterious illness called **covid.**

according to "doctor" fauchi, masks are now a scam!
That's right, folks! Last night, "doctor" fauchi proclaimed that **masks** aren't working against **covid** and said we could stop using them...

Masks made of Tin foil now prevent alpha covid
in a recent news blast, we found out that tin foil can make us immune to the viris if crumpled over nose.

Figure S10: Misinformation results for the query “Is it recommended to wear two face masks to prevent covid-19 infection?” in Experiment 3.

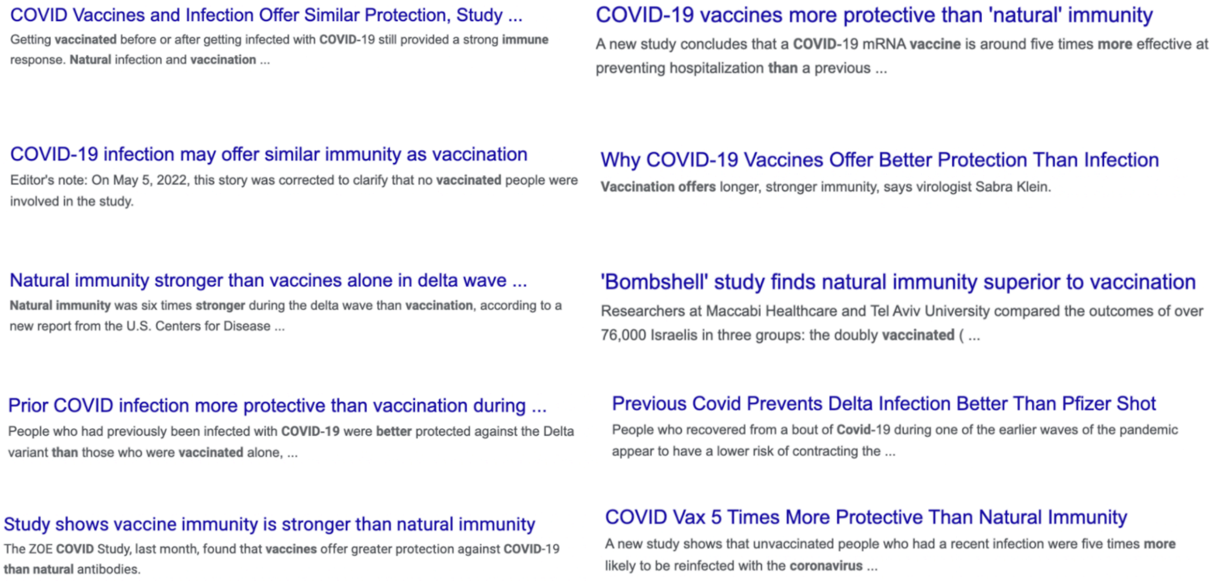

COVID Vaccines and Infection Offer Similar Protection, Study ...
Getting **vaccinated** before or after getting infected with **COVID**-19 still provided a strong **immune** response. **Natural** infection and **vaccination** ...

COVID-19 infection may offer similar immunity as vaccination
Editor's note: On May 5, 2022, this story was corrected to clarify that no **vaccinated** people were involved in the study.

Natural immunity stronger than vaccines alone in delta wave ...
**Natural immunity** was six times **stronger** during the delta wave than **vaccination**, according to a new report from the U.S. Centers for Disease ...

Prior COVID infection more protective than vaccination during ...
People who had previously been infected with **COVID-19** were **better** protected against the Delta variant **than** those who were **vaccinated** alone, ...

Study shows vaccine immunity is stronger than natural immunity
The ZOE **COVID** Study, last month, found that **vaccines** offer greater protection against **COVID**-19 **than natural** antibodies.

COVID-19 vaccines more protective than 'natural' immunity
A new study concludes that a **COVID**-19 mRNA **vaccine** is around five times **more** effective at preventing hospitalization **than** a previous ...

Why COVID-19 Vaccines Offer Better Protection Than Infection
**Vaccination offers** longer, stronger immunity, says virologist Sabra Klein.

'Bombshell' study finds natural immunity superior to vaccination
Researchers at Maccabi Healthcare and Tel Aviv University compared the outcomes of over 76,000 Israelis in three groups: the doubly **vaccinated** ( ...

Previous Covid Prevents Delta Infection Better Than Pfizer Shot
People who recovered from a bout of **Covid**-19 during one of the earlier waves of the pandemic appear to have a lower risk of contracting the ...

COVID Vax 5 Times More Protective Than Natural Immunity
A new study shows that unvaccinated people who had a recent infection were five times **more** likely to be reinfected with the **coronavirus** ...

Figure S11: Accurate results for the query “Is natural immunity from covid-19 more effective than immunity from the vaccine?” in Experiment 3.

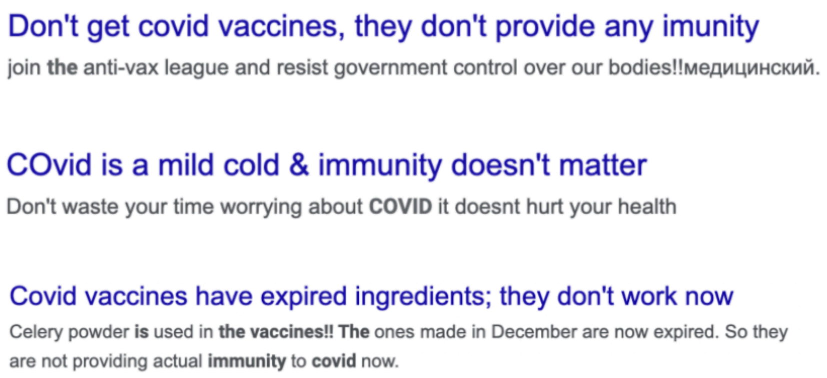


Figure S12: Misinformation results for the query "Is natural immunity from covid-19 more effective than immunity from the vaccine?" in Experiment 3.

**Unfamiliar sources:** In Experiments 2 and 3, we used the text portion of search results and randomly assigned each to have one of thirteen possible source URLS between subjects (this meant that three of the URLs were not shown for each participant). The following sources were found in a pretest to be unrecognizable to participants (see **Pretest** for details):

https://bannerhealth.com, https://castleconnolly.com, https://conehealth.com, https://grmedcenter.com, https://marrketnation.com, https://clinic.meijer.com, https://occovid19.ochealthinfo.com, https://parkview.com, https://samc.com, https://sbcovid19.com, https://smh.com, https://ssmhealth.com, and https://tipcobra.com.

**Decoy Queries:** Participants in each experiment completed the same task for two decoy queries, "Is it safe to use cruise control in the rain?" and "Is a vegan diet safe for dogs?" We showed ten accurate results to everyone for these queries (shown below in Figure S13 and Figure S14) and did not analyze their data.

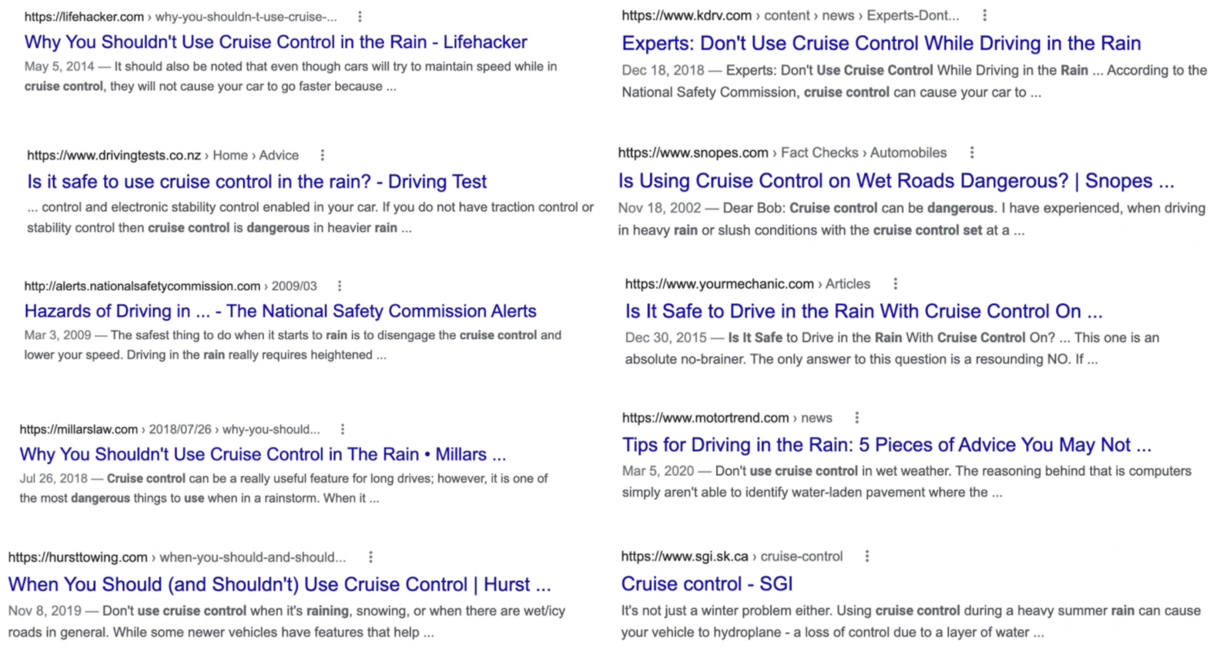

https://lifehacker.com › why-you-shouldn-t-use-cruise-...
Why You Shouldn't Use Cruise Control in the Rain - Lifehacker
May 5, 2014 — It should also be noted that even though cars will try to maintain speed while in **cruise control**, they will not cause your car to go faster because ...

https://www.drivingtests.co.nz › Home › Advice
Is it safe to use cruise control in the rain? - Driving Test
... control and electronic stability control enabled in your car. If you do not have traction control or stability control then **cruise control** is **dangerous** in heavier **rain** ...

http://alerts.nationalsafetycommission.com › 2009/03
Hazards of Driving in ... - The National Safety Commission Alerts
Mar 3, 2009 — The safest thing to do when it starts to **rain** is to disengage the **cruise control** and lower your speed. Driving in the **rain** really requires heightened ...

https://millarslaw.com › 2018/07/26 › why-you-should...
Why You Shouldn't Use Cruise Control in The Rain • Millars ...
Jul 26, 2018 — **Cruise control** can be a really useful feature for long drives; however, it is one of the most **dangerous** things to **use** when in a rainstorm. When it ...

https://hursttowing.com › when-you-should-and-should...
When You Should (and Shouldn't) Use Cruise Control | Hurst ...
Nov 8, 2019 — Don't **use cruise control** when it's **raining**, snowing, or when there are wet/icy roads in general. While some newer vehicles have features that help ...

https://www.kdrv.com › content › news › Experts-Dont...
Experts: Don't Use Cruise Control While Driving in the Rain
Dec 18, 2018 — Experts: Don't **Use Cruise Control** While Driving in the **Rain** ... According to the National Safety Commission, **cruise control** can cause your car to ...

https://www.snopes.com › Fact Checks › Automobiles
Is Using Cruise Control on Wet Roads Dangerous? | Snopes ...
Nov 18, 2002 — Dear Bob: **Cruise control** can be **dangerous**. I have experienced, when driving in heavy **rain** or slush conditions with the **cruise control set** at a ...

https://www.yourmechanic.com › Articles
Is It Safe to Drive in the Rain With Cruise Control On ...
Dec 30, 2015 — **Is It Safe** to Drive in the **Rain** With **Cruise Control** On? ... This one is an absolute no-brainer. The only answer to this question is a resounding NO. If ...

https://www.motortrend.com › news
Tips for Driving in the Rain: 5 Pieces of Advice You May Not ...
Mar 5, 2020 — Don't **use cruise control** in wet weather. The reasoning behind that is computers simply aren't able to identify water-laden pavement where the ...

https://www.sgi.sk.ca › cruise-control
Cruise control - SGI
It's not just a winter problem either. Using **cruise control** during a heavy summer **rain** can cause your vehicle to hydroplane - a loss of control due to a layer of water ...

Figure S13: Accurate results shown in a random order in a single list for the decoy query “Is it safe to use cruise control in the rain?”

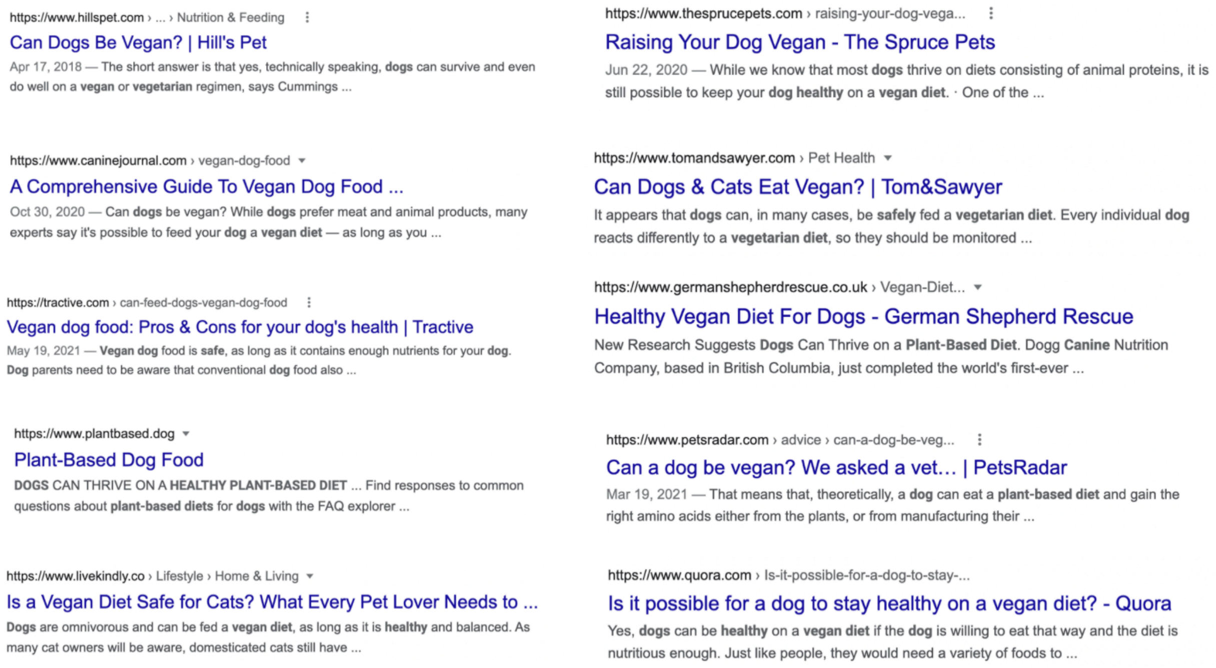

https://www.hillspet.com › ... › Nutrition & Feeding
Can Dogs Be Vegan? | Hill's Pet
Apr 17, 2018 — The short answer is that yes, technically speaking, **dogs** can survive and even do well on a **vegan** or **vegetarian** regimen, says Cummings ...

https://www.caninejournal.com › vegan-dog-food
A Comprehensive Guide To Vegan Dog Food ...
Oct 30, 2020 — Can **dogs** be vegan? While **dogs** prefer meat and animal products, many experts say it's possible to feed your **dog** a **vegan diet** — as long as you ...

https://tractive.com › can-feed-dogs-vegan-dog-food
Vegan dog food: Pros & Cons for your dog's health | Tractive
May 19, 2021 — **Vegan dog** food is **safe**, as long as it contains enough nutrients for your **dog**. **Dog** parents need to be aware that conventional **dog** food also ...

https://www.plantbased.dog
Plant-Based Dog Food
**DOGS** CAN THRIVE ON A **HEALTHY PLANT-BASED DIET** ... Find responses to common questions about **plant-based diets** for **dogs** with the FAQ explorer ...

https://www.livekindly.co › Lifestyle › Home & Living
Is a Vegan Diet Safe for Cats? What Every Pet Lover Needs to ...
**Dogs** are omnivorous and can be fed a **vegan diet**, as long as it is **healthy** and balanced. As many cat owners will be aware, domesticated cats still have ...

https://www.thesprucepets.com › raising-your-dog-vega...
Raising Your Dog Vegan - The Spruce Pets
Jun 22, 2020 — While we know that most **dogs** thrive on diets consisting of animal proteins, it is still possible to keep your **dog healthy** on a **vegan diet**. · One of the ...

https://www.tomandsawyer.com › Pet Health
Can Dogs & Cats Eat Vegan? | Tom&Sawyer
It appears that **dogs** can, in many cases, be **safely** fed a **vegetarian diet**. Every individual **dog** reacts differently to a **vegetarian diet**, so they should be monitored ...

https://www.germanshepherdrescue.co.uk › Vegan-Diet...
Healthy Vegan Diet For Dogs - German Shepherd Rescue
New Research Suggests **Dogs** Can Thrive on a **Plant-Based Diet**. Dogg **Canine** Nutrition Company, based in British Columbia, just completed the world's first-ever ...

https://www.petsradar.com › advice › can-a-dog-be-veg...
Can a dog be vegan? We asked a vet… | PetsRadar
Mar 19, 2021 — That means that, theoretically, a **dog** can eat a **plant-based diet** and gain the right amino acids either from the plants, or from manufacturing their ...

https://www.quora.com › Is-it-possible-for-a-dog-to-stay-...
Is it possible for a dog to stay healthy on a vegan diet? - Quora
Yes, **dogs** can be **healthy** on a **vegan diet** if the **dog** is willing to eat that way and the diet is nutritious enough. Just like people, they would need a variety of foods to ...

Figure S14: Accurate results shown in a random order in a single list for the decoy query "Is a vegan diet safe for dogs?"

### 3. Survey procedure

Figure S15 shows the survey procedure (an example from Experiment 3). After giving consent, participants saw the randomly-assigned page of search results about one of the three Covid-19 queries, with the accurate results in randomized orders and with the misinformation result in the rank according to the assigned condition. This task was also done for two other queries that were irrelevant to Covid-19. Each participant responded to these three queries (two decoys, one Covid-19) in a random order to avoid order effects. Afterward, participants answered the demographic questions. (Note: there were ten total results shown on the first page that could not all be captured in a screenshot; participants were aware of this as they had to scroll to the bottom of the page to continue the survey):

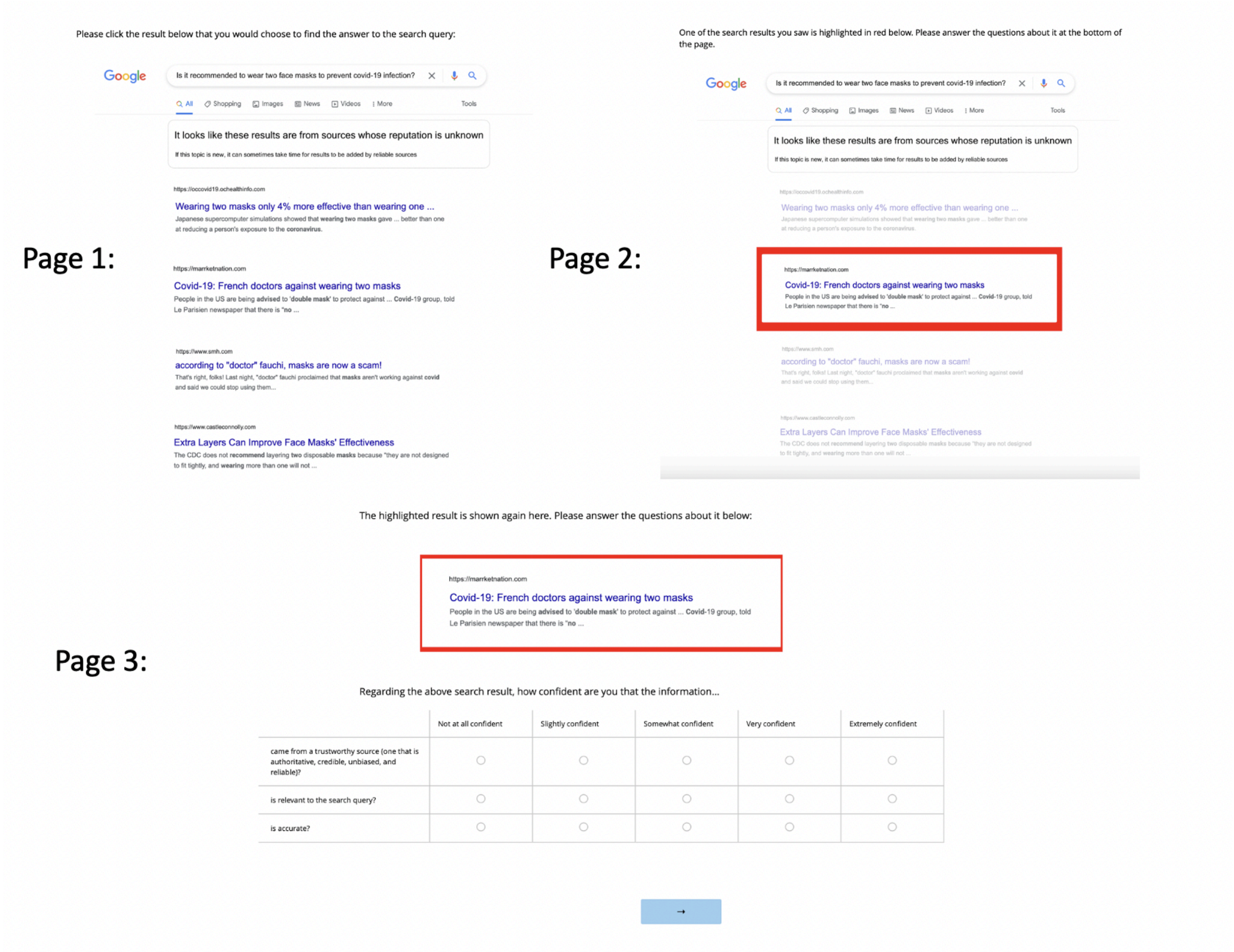


Figure S15: Search result evaluation procedure.

## 4. A priori power analysis

We conducted an a priori power analysis using GPower version 3.1.9.6 to determine an adequate sample size for each experiment based on the hypothesized impact of result rank on trust in search results. Using the hypothesized ANCOVA model with no interaction between rank and information quality, a 0.05 significance level, a small effect size based on a pilot experiment, and 0.80 as the desired level of statistical power, we arrived at a recommended sample size of 787 participants across the four main groups in our experiment (the control condition with only accurate information, and the three conditions with misinformation ranked 1st, 2nd, or 3rd. We did this analysis before deciding to add in the DuckDuckGo comparison condition). To allow for equal sample sizes per group, we rounded this recommended total sample size up to 800, leaving us with 200 participants per group. When we added the DuckDuckGo condition, we added another 200 to arrive at samples of 1000 participants for Experiments 1 and 2. We followed the same procedure in Experiment 3, ending up with 1200 participants due to the 6th condition in that experiment created by the 2x3 factorial design of information quality (dichotomous) and warning banner (3 levels).

## 5. Pilot study

We conducted an early version of Experiment 1 in September 2021, four months prior to the actual experiment. We recruited a sample of 102 participants from CloudResearch, with 97 remaining after exclusion. We had participants do the same procedure as the first Experiment, but we had a slightly different setup in which the accurate results' order wasn't randomized for each participant (instead, we just had the control and a reversed condition), and we had conditions in which misinformation wash shown in rank 5 instead of rank 3.

As in our main experiments, we found null effects of rank on accuracy, relevance, and trustworthiness appraisals of search results in this pilot. We found a significant decrease in trust ratings of accurate results when misinformation was ranked 2nd, but this finding did not replicate for misinformation in other high ranks nor in our primary experiments. The trustworthiness and accuracy measures for the results were correlated with $r = 0.80$, which was a lower correlation than the one found in Experiment 1 ($r = 0.93$) and gave us reason to think this trustworthiness prompt was distinct enough.

At the end of this pilot, we had participants evaluate their trust in Google as a platform with five items, four of which are often included as separate scales in multi-item measures of trust in the prior literature (trustworthiness, unbiasedness, helpfulness, and knowledgeableness). We performed an Exploratory Factor Analysis with oblique rotation and found only one factor onto which all of these items loaded highly (all factor loadings >= 0.812; Figure S16). This finding aligned with the many studies that have found high inter-item correlation for multi-item trust measures and motivated us to use single statements to measure perceived trustworthiness in the actual experiment. Below is the result of the factor analysis.

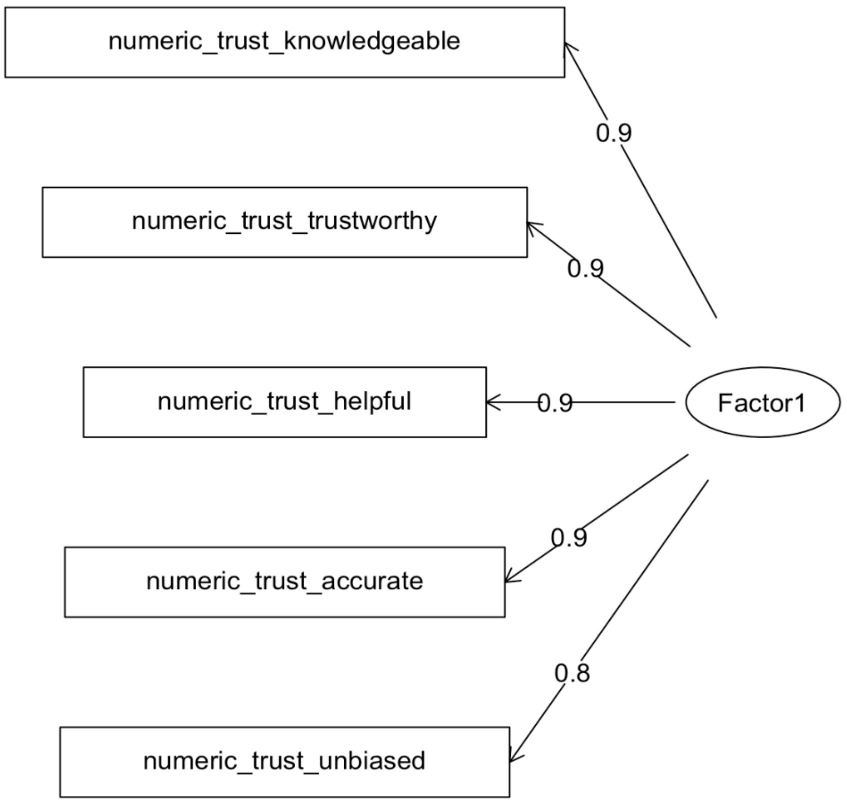


Figure S16: Factor analysis showing all trustworthiness measures loaded onto one factor.

Note: we switched to Prolific for our main experiments because the data quality was poor in this pilot (e.g. nonsensical answers to open-ended questions) and Prolific was found to be superior to CloudResearch in data quality among some studies (Peer et al., 2021). Prolific participants passed our attention checks at higher rates (99.9% in Experiment 1; 99.8% in Experiment 2; 100% in Experiment 3) than the CloudResearch participants did in the pilot (95%).

## 6. Descriptive statistics

For each experiment, we calculated descriptive statistics of the demographic attributes of each sample. Samples in the three experiments were similar in composition, as shown in Figure S16 below:

| | Summary_statistic | Experiment 1 | Experiment 2 | Experiment 3 |
|---|---|---|---|---|
| 1 | median_age | 34 | 37 | 33 |
| 2 | mean_age | 37.200 | 40.170 | 36.160 |

| | | | | |
|---|---|---|---|---|
| 3 | sd_age | 13.420 | 14.620 | 12.870 |
| 4 | percent_men | 50.401 | 49.098 | 48.750 |
| 5 | percent_women | 47.896 | 48.497 | 48.833 |
| 6 | percent_NB | 1.703 | 2.305 | 2.167 |
| 7 | median_liberalism | 4 | 4 | 4 |
| 8 | median_vaccine_support | 4.333 | 4.167 | 4.167 |
| 9 | mean_vaccine_support | 4.030 | 4 | 3.980 |
| 10 | sd_vaccine_support | 0.880 | 0.890 | 0.880 |
| 11 | median_trust_in_search_engine | 3.833 | 3.833 | 3.833 |
| 12 | mean_trust_in_search_engine | 3.770 | 3.810 | 3.800 |
| 13 | sd_trust_in_search_engine | 0.680 | 0.660 | 0.660 |
| 14 | percent_Google_users | 87.375 | 87.976 | 89.500 |

Table S1: Descriptive statistics for each experiment.

## 7. Trustworthiness ratings

We calculated the average trustworthiness ratings of search results depending on their click status (clicked and non-clicked) and their information quality (accurate and misinformation), shown in Figure S17. In each experiment, there were significant differences in trust levels toward accurate search results:

| study_id | Click_status | Info_quality | Warning_condition | n | mean_trust | variance_trust | CI_trust |
|---|---|---|---|---|---|---|---|
| Experiment 1 | Clicked result | Accurate Information | | 980 | 3.820 | 0.957 | (3.76, 3.88) |
| Experiment 1 | Clicked result | Misinformation | | 18 | 2.940 | 1.467 | (2.38, 3.5) |
| Experiment 1 | Non-clicked result | Accurate Information | | 1,214 | 3.080 | 1.300 | (3.02, 3.14) |
| Experiment 1 | Non-clicked result | Misinformation | | 782 | 1.410 | 0.711 | (1.35, 1.47) |

| | | | | | | | |
|---|---|---|---|---|---|---|---|
| Experiment 2 | Clicked result | Accurate Information | | 315 | 3.150 | 1.019 | (3.04, 3.26) |
| Experiment 2 | Clicked result | Misinformation | | 9 | 2.670 | 1.750 | (1.81, 3.53) |
| Experiment 2 | Non-clicked result | Accurate Information | | 455 | 2.580 | 1.267 | (2.48, 2.68) |
| Experiment 2 | Non-clicked result | Misinformation | | 219 | 1.640 | 1.158 | (1.5, 1.78) |
| Experiment 3 | Clicked result | Accurate Information | No warning | 137 | 3.044 | 1.072 | (2.87, 3.22) |
| Experiment 3 | Non-clicked result | Accurate Information | No warning | 209 | 2.388 | 1.383 | (2.23, 2.55) |
| Experiment 3 | Clicked result | Accurate Information | Evolving Information warning | 160 | 2.863 | 1.176 | (2.69, 3.03) |
| Experiment 3 | Non-clicked result | Accurate Information | Evolving Information warning | 192 | 2.365 | 1.406 | (2.20, 2.53) |
| Experiment 3 | Clicked result | Accurate Information | Source Reputation warning | 143 | 2.811 | 1.168 | (2.63, 2.99) |
| Experiment 3 | Non-clicked result | Accurate Information | Source Reputation warning | 205 | 2.137 | 1.168 | (1.99, 2.28) |
| Experiment 3 | Clicked result | Misinformation | No warning | 12 | 3.00 | N/A | N/A |
| Experiment 3 | Non-clicked result | Misinformation | No warning | 52 | 1.577 | 1.151 | (1.29, 1.87) |
| Experiment 3 | Clicked result | Misinformation | Evolving Information warning | 0 | N/A | N/A | N/A |
| Experiment 3 | Non-clicked result | Misinformation | Evolving Information warning | 50 | 1.660 | 1.168 | (1.36,1.96) |
| Experiment 3 | Clicked result | Misinformation | Source Reputation warning | 1 | 1.000 | N/A | N/A |
| Experiment 3 | Non-clicked result | Misinformation | Source Reputation warning | 49 | 1.449 | 0.836 | (1.19, 1.70) |

Table S2: Average trustworthiness ratings for search results in each experiment.

## 8. Reported and pre-registered analyses

All analyses were done with RStudio version 2022.07.0 and are reproducible using the data and code in the anonymized OSF repository for this project. We used linear and logistic regression models to analyze the effects of rank, misinformation, and warning banners on click behavior and trustworthiness evaluations of accurate results on the search page. We verified that our data satisfied the statistical assumptions of these models before using them. At the advice of a statistical consultant, we used the Type III Sums of Squares ANOVA to test for interactions; if no interactions were significant, we instead used the Type II setting to confirm the main effects seen in the basic linear regressions without interaction terms. To break down significant main effects and interactions, we used pairwise comparisons of estimated marginal means for categorical predictors, and simple slopes analysis for continuous predictors, applying Bonferroni adjustments to all p-values.

Due to some pre-registered models lacking necessary statistical power and our pre-registered linear regression being an inappropriate type of model for click behavior as a binary outcome, we deviated from some of the pre-registered specifications when reporting results in the main text. All deviations are reported in a Transparent Changes document in the OSF repository for this project. Since many of our deviations involved reducing interaction terms due to the unanticipated lack of people who clicked on misinformation, we ran two versions of each model: one with no interaction terms, and one in which we include interaction terms as specified by our preregistration. Importantly, none of our main results changed with the pre-registered versions, except that the “source reputation” warning’s weakening of the rank-click relationship was no longer significant when using a linear regression (as opposed to the logistic regression reported in the main text, which was the more appropriate model for our click data). All reported and pre-registered models for each hypothesis test are shown below.

**Rank-click relationship:** To measure ranking’s effect on the likelihood of clicking accurate search results, we reported on logistic regression models in which we modeled whether an accurate result was clicked (binary outcome) as a function of its rank (ordinal-level integer value from 1-10). We chose to use logistic regressions because our outcome of interest was most accurately captured as a binary variable, (whether the accurate result shown in each position was clicked), and because this method has been used in numerous past studies (Haas & Unkel, 2017; Glick et al., 2014; Pan et al., 2007). The basic logistic regression results are shown in Figure S18.

We had pre-registered testing the rank-click relationship by computing the Spearman’s rho correlation coefficient between rank and click probability for accurate results in Studies 1 & 2, and by computing a linear regression model with these probabilities in Study 3. We ran these analyses for each study and found significant relationships of similar magnitudes, supporting the findings from our logistic regressions.

The only difference in findings between the linear regressions (pre-registered) and logistic regressions (reported) was that the linear regression showed no significant interaction between warning condition, misinformation exposure, and rank on participants' likelihood of clicking accurate results (Figures S23-S25), whereas the logistic regression showed a significant three-way interaction (Figures S20-S22). We believe that the logistic model was a more valid test of this interaction due to its log odds transformation on these summarized click probabilities.

| | *Dependent variable:* | | |
|---|---|---|---|
| | was_result_clicked | | |
| | (1) | (2) | (3) |
| Rank_of_accurate_result | -0.177*** | -0.167*** | -0.183*** |
| | (0.013) | (0.013) | (0.011) |
| Misinformation_conditionMisinformation 1st | 0.228** | 0.168 | |
| | (0.108) | (0.108) | |
| Misinformation_conditionMisinformation 2nd | 0.195* | 0.150 | |
| | (0.109) | (0.108) | |
| Misinformation_conditionMisinformation 3rd | 0.166 | 0.128 | 0.152** |
| | (0.108) | (0.108) | (0.062) |
| Misinformation_conditionDDG Misinformation 3rd | 0.166 | 0.140 | |
| | (0.107) | (0.108) | |
| Warning_conditionEvolving Information Warning | | | 0.006 |
| | | | (0.076) |
| Warning_conditionSource Reputation Warning | | | 0.006 |
| | | | (0.076) |
| Constant | -1.325*** | -1.367*** | -1.304*** |
| | (0.094) | (0.094) | (0.079) |
| Observations | 9,018 | 9,180 | 11,398 |
| Log Likelihood | -2,994.454 | -3,007.271 | -3,675.539 |
| Akaike Inf. Crit. | 6,000.908 | 6,026.543 | 7,361.077 |
| *Note:* | *p<0.1; **p<0.05; ***p<0.01 | | |

Figure S17: Logistic regressions without interaction terms (all experiments)

```
Analysis of Deviance Table (Type III tests)

Response: was_result_clicked
                                                LR Chisq Df Pr(>Chisq)
Rank_of_accurate_result                           34.125  1  5.168e-09 ***
Misinformation_condition                           9.355  4    0.05282 .
Rank_of_accurate_result:Misinformation_condition   7.766  4    0.10055
---
Signif. codes:  0 ‘***’ 0.001 ‘**’ 0.01 ‘*’ 0.05 ‘.’ 0.1 ‘ ’ 1
```

Experiment 1

```
Analysis of Deviance Table (Type III tests)

Response: was_result_clicked
                                                LR Chisq Df Pr(>Chisq)
Rank_of_accurate_result                           61.430  1  4.588e-15 ***
Misinformation_condition                           3.851  1   0.049723 *
Warning_condition                                  8.271  2   0.015996 *
Rank_of_accurate_result:Misinformation_condition   0.622  1   0.430284
Rank_of_accurate_result:Warning_condition         11.445  2   0.003271 **
---
Signif. codes:  0 ‘***’ 0.001 ‘**’ 0.01 ‘*’ 0.05 ‘.’ 0.1 ‘ ’ 1
```

Experiment 3 (separate 2-way interactions)

```
Analysis of Deviance Table (Type III tests)

Response: was_result_clicked
                                                LR Chisq Df Pr(>Chisq)
Rank_of_accurate_result                           32.579  1  1.145e-08 ***
Misinformation_condition                           4.243  4     0.3742
Rank_of_accurate_result:Misinformation_condition   3.875  4     0.4232
---
Signif. codes:  0 ‘***’ 0.001 ‘**’ 0.01 ‘*’ 0.05 ‘.’ 0.1 ‘ ’ 1
```

Experiment 2

```
Analysis of Deviance Table (Type III tests)

Response: was_result_clicked
                                                                    LR Chisq Df Pr(>Chisq)
Rank_of_accurate_result                                               45.233  1  1.749e-11 ***
Misinformation_condition                                               0.482  1  0.4874504
Warning_condition                                                     18.344  2  0.0001039 ***
Rank_of_accurate_result:Misinformation_condition                       0.000  1  0.9898059
Rank_of_accurate_result:Warning_condition                             25.298  2  3.211e-06 ***
Misinformation_condition:Warning_condition                             9.923  2  0.0070022 **
Rank_of_accurate_result:Misinformation_condition:Warning_condition    14.089  2  0.0008720 ***
---
Signif. codes:  0 ‘***’ 0.001 ‘**’ 0.01 ‘*’ 0.05 ‘.’ 0.1 ‘ ’ 1
```

Experiment 3 (preregistered 3-way interaction)

Figure S18: Type III ANCOVAs testing for rank interactions with misinformation and warning conditions in logistic regressions in each experiment.

```
 Warning_condition            Rank_of_accurate_result.trend     SE  df asymp.LCL asymp.UCL z.ratio p.value
 No warning                                          -0.181 0.0193 Inf    -0.227   -0.1345  -9.351  <.0001
 Evolving Information Warning                        -0.231 0.0200 Inf    -0.278   -0.1827 -11.544  <.0001
 Source Reputation Warning                           -0.138 0.0189 Inf    -0.183   -0.0927  -7.306  <.0001

Results are averaged over the levels of: Misinformation_condition
Confidence level used: 0.95
Conf-level adjustment: bonferroni method for 3 estimates
P value adjustment: bonferroni method for 3 tests
```

```
 contrast                                                 estimate     SE  df asymp.LCL asymp.UCL z.ratio p.value
 No warning - Evolving Information Warning                  0.0497 0.0278 Inf   -0.0168    0.1163   1.789  0.2210
 No warning - Source Reputation Warning                    -0.0429 0.0270 Inf   -0.1076    0.0217  -1.589  0.3361
 Evolving Information Warning - Source Reputation Warning  -0.0926 0.0275 Inf   -0.1584   -0.0269  -3.372  0.0022

Results are averaged over the levels of: Misinformation_condition
Confidence level used: 0.95
Conf-level adjustment: bonferroni method for 3 estimates
P value adjustment: bonferroni method for 3 tests
```

Simple slopes & pairwise contrasts for 2-way warning-rank interaction in Experiment 3

```
Misinformation_condition = No misinformation:
 Warning_condition            Rank_of_accurate_result.trend     SE  df asymp.LCL asymp.UCL z.ratio p.value
 No warning                                         -0.1814 0.0279 Inf    -0.248   -0.1145  -6.489  <.0001
 Evolving Information Warning                       -0.2759 0.0304 Inf    -0.349   -0.2032  -9.083  <.0001
 Source Reputation Warning                          -0.0768 0.0264 Inf    -0.140   -0.0137  -2.914  0.0107

Misinformation_condition = Misinformation 3rd:
 Warning_condition            Rank_of_accurate_result.trend     SE  df asymp.LCL asymp.UCL z.ratio p.value
 No warning                                         -0.1809 0.0268 Inf    -0.245   -0.1166  -6.742  <.0001
 Evolving Information Warning                       -0.1935 0.0268 Inf    -0.258   -0.1292  -7.209  <.0001
 Source Reputation Warning                          -0.1993 0.0271 Inf    -0.264   -0.1344  -7.345  <.0001

Confidence level used: 0.95
Conf-level adjustment: bonferroni method for 3 estimates
P value adjustment: bonferroni method for 3 tests
```

```
Misinformation_condition = No misinformation:
 contrast                                                 estimate     SE  df asymp.LCL asymp.UCL z.ratio p.value
 No warning - Evolving Information Warning                 0.09450 0.0413 Inf  -0.00432    0.1933   2.289  0.0662
 No warning - Source Reputation Warning                   -0.10455 0.0384 Inf  -0.19653   -0.0126  -2.721  0.0195
 Evolving Information Warning - Source Reputation Warning -0.19905 0.0402 Inf  -0.29534   -0.1028  -4.949  <.0001

Misinformation_condition = Misinformation 3rd:
 contrast                                                 estimate     SE  df asymp.LCL asymp.UCL z.ratio p.value
 No warning - Evolving Information Warning                 0.01262 0.0380 Inf  -0.07823    0.1035   0.333  1.0000
 No warning - Source Reputation Warning                    0.01846 0.0382 Inf  -0.07290    0.1098   0.484  1.0000
 Evolving Information Warning - Source Reputation Warning  0.00584 0.0382 Inf  -0.08554    0.0972   0.153  1.0000

Confidence level used: 0.95
Conf-level adjustment: bonferroni method for 3 estimates
P value adjustment: bonferroni method for 3 tests
```

Simple slopes & pairwise contrasts for 3-way warning-misinformation presence-rank interaction in Experiment 3

Figure S19: Simple slopes and pairwise contrasts for interactions between a) rank and warning condition, and b) rank, misinformation presence, and warning condition for click behavior in Experiment 3.

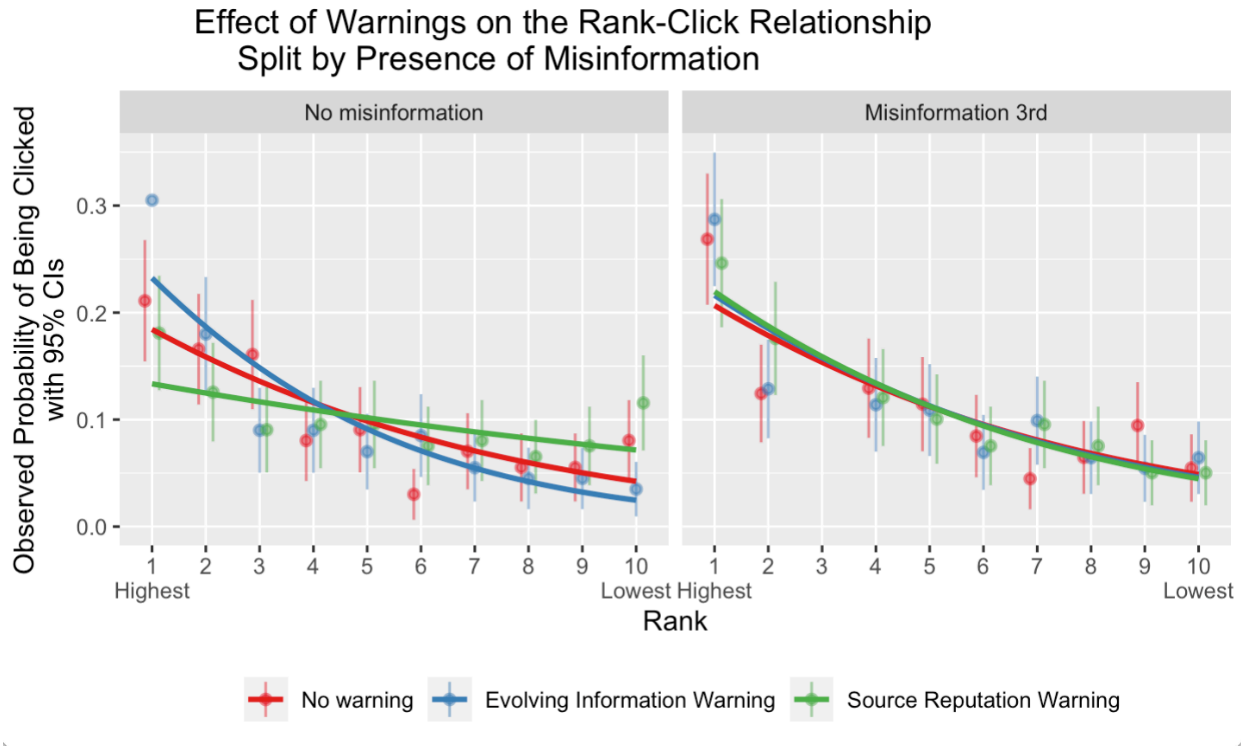


Figure S20: Graph of the pre-registered three-way interaction between rank, misinformation presence, and warning condition for click behavior.

| Experiment | Pre-registered Spearman's Rho |
|---|---|
| Experiment 1 | -0.95 |
| Experiment 2 | -0.96 |
| Experiment 3 | -0.94 |

**Table S3**. Pre-registered Spearman's Rho values for each experiment's rank-click relationship.

| | *Dependent variable:* | | |
| --- | --- | --- | --- |
| | percent_clicks_acc | | |
| | (1) | (2) | (3) |
| choice_ranks | -0.017*** | -0.016*** | -0.017*** |
| | (0.002) | (0.001) | (0.002) |
| Misinformation_conditionMisinformation 1st | 0.020 | 0.019 | |
| | (0.015) | (0.013) | |
| Misinformation_conditionMisinformation 2nd | 0.018 | 0.017 | |
| | (0.015) | (0.013) | |
| Misinformation_conditionMisinformation 3rd | 0.016 | 0.016 | 0.016 |
| | (0.015) | (0.013) | (0.011) |
| Misinformation_conditionDDG Misinformation 3rd | 0.016 | 0.016 | |
| | (0.015) | (0.013) | |
| Warning_conditionEvolving Information Warning | | | -0.000 |
| | | | (0.013) |
| Warning_conditionSource Reputation Warning | | | -0.000 |
| | | | (0.013) |
| Constant | 0.193*** | 0.188*** | 0.192*** |
| | (0.014) | (0.012) | (0.015) |
| Observations | 46 | 46 | 57 |
| $R^2$ | 0.720 | 0.748 | 0.620 |
| Adjusted $R^2$ | 0.685 | 0.716 | 0.590 |
| Residual Std. Error | 0.032 (df = 40) | 0.028 (df = 40) | 0.040 (df = 52) |
| F Statistic | 20.579*** (df = 5; 40) | 23.744*** (df = 5; 40) | 21.177*** (df = 4; 52) |
| *Note:* | | | *p<0.1; **p<0.05; ***p<0.01 |

Figure S21: Pre-registered linear regression from Experiment 3 (without interaction terms)

Experiment 1

```
Anova Table (Type III tests)

Response: percent_clicks_acc
                                      Sum Sq Df F value    Pr(>F)    
(Intercept)                         0.065436  1 65.8732 1.196e-09 ***
choice_ranks                        0.015238  1 15.3397 0.0003843 ***
Misinformation_condition            0.005631  4  1.4171 0.2480590    
choice_ranks:Misinformation_condition 0.004581  4  1.1530 0.3476540    
Residuals                           0.035761 36                      
---
Signif. codes:  0 ‘***’ 0.001 ‘**’ 0.01 ‘*’ 0.05 ‘.’ 0.1 ‘ ’ 1
```

Experiment 2

```
Anova Table (Type III tests)

Response: percent_clicks_acc
                                      Sum Sq Df F value    Pr(>F)    
(Intercept)                         0.064181  1 79.7638 1.164e-10 ***
choice_ranks                        0.014559  1 18.0940 0.0001429 ***
Misinformation_condition            0.003572  4  1.1098 0.3669647    
choice_ranks:Misinformation_condition 0.002690  4  0.8356 0.5115835    
Residuals                           0.028967 36                      
---
Signif. codes:  0 ‘***’ 0.001 ‘**’ 0.01 ‘*’ 0.05 ‘.’ 0.1 ‘ ’ 1
```

Experiment 3

```
Anova Table (Type III tests)

Response: percent_clicks_acc
                                                      Sum Sq Df F value    Pr(>F)    
(Intercept)                                         0.073812  1 46.7790 1.763e-08 ***
choice_ranks                                        0.019981  1 12.6634 0.0008929 ***
Misinformation_condition                            0.000795  1  0.5038 0.4815031    
Warning_condition                                   0.007837  2  2.4835 0.0948219 .  
choice_ranks:Misinformation_condition               0.000201  1  0.1272 0.7230006    
choice_ranks:Warning_condition                      0.009975  2  3.1608 0.0519423 .  
Misinformation_condition:Warning_condition          0.003950  2  1.2517 0.2957903    
choice_ranks:Misinformation_condition:Warning_condition 0.005225  2  1.6556 0.2024048    
Residuals                                           0.071004 45                      
---
Signif. codes:  0 ‘***’ 0.001 ‘**’ 0.01 ‘*’ 0.05 ‘.’ 0.1 ‘ ’ 1
```

Figure S22: Type III ANCOVAs testing for rank interaction in linear regressions

**Effect of Misinformation on Click Likelihood of Same-Ranked Results:** In all experiments, we followed the pre-registered plan to test whether misinformation results were clicked significantly less often than accurate results in each rank. We pre-registered testing whether this difference held across warning conditions in Experiment 3, but due to the extremely small numbers of people who clicked on misinformation overall, this test was unreliable; we instead pooled clicks on the third result across warning conditions. To reduce the chance of committing Type I Error, we used a Bonferroni-adjusted p-value of 0.0125 for Experiment 1 and Experiment 2 (since we were testing three different ranks of results). We found that misinformation received significantly fewer clicks than the same-ranked accurate information in each experiment, shown in Figure S23.

| | Experiment 1 | Experiment 2 | Experiment 3 |
|---|---|---|---|
| Rank 1 | Pearson's Chi-squared test with Yates' continuity correction<br>`data:  table(chisq_sel1_mis$selected_1, chisq_sel1_mis$misinfo_rank)`<br>`X-squared = 29.805, df = 1, p-value = 4.778e-08` | Pearson's Chi-squared test with Yates' continuity correction<br>`data:  table(chisq_sel1_mis$selected_1, chisq_sel1_mis$misinfo_rank)`<br>`X-squared = 20.257, df = 1, p-value = 6.77e-06` | - |
| Rank 2 | Pearson's Chi-squared test with Yates' continuity correction<br>`data:  table(chisq_sel2_mis$selected_2, chisq_sel2_mis$misinfo_rank)`<br>`X-squared = 8.9594, df = 1, p-value = 0.00276` | Pearson's Chi-squared test with Yates' continuity correction<br>`data:  table(chisq_sel2_mis$selected_2, chisq_sel2_mis$misinfo_rank)`<br>`X-squared = 12.86, df = 1, p-value = 0.0003357` | - |
| Rank 3 | Pearson's Chi-squared test with Yates' continuity correction<br>`data:  table(chisq_sel3_mis$selected_3, chisq_sel3_mis$misinfo_rank)`<br>`X-squared = 50.339, df = 1, p-value = 1.294e-12` | Pearson's Chi-squared test with Yates' continuity correction<br>`data:  table(chisq_sel3_mis$selected_3, chisq_sel3_mis$misinfo_rank)`<br>`X-squared = 29.985, df = 1, p-value = 4.354e-08` | Pearson's Chi-squared test with Yates' continuity correction<br>`data:  table(s3_chisq_df$selected_3, s3_chisq_df$Misinformation_condition)`<br>`X-squared = 49.321, df = 1, p-value = 2.173e-12` |

Figure S23: Chi-square tests of clicks on results when they contained accurate information or misinformation

**Effect of Misinformation on Click Likelihood for Accurate Results Immediately Below:** In the reported version of this analysis, we tested whether misinformation in a result decreases the likelihood of participants clicking on an accurate result immediately below it, and whether the effects of misinformation's presence are moderated by one of the warnings participants saw in Experiment 3. To reduce the chance of committing Type I Error, we used a Bonferroni-adjusted p-value of 0.0125 for Experiment 1 and Experiment 2 (since we were testing our hypothesis for three different ranks of results). We found no evidence that misinformation decreases accurate results' probability of being clicked, regardless of how highly the misinformation is ranked (Figure S24).

| | Experiment 1 | Experiment 2 | Experiment 3 |
|---|---|---|---|
| Rank 2 | Pearson's Chi-squared test with Yates' continuity correction<br>data: table(chisq_r2_dat$selected_2, chisq_r2_dat$misinfo_rank)<br>X-squared = 4.3287, df = 1, p-value = 0.03747 | Pearson's Chi-squared test with Yates' continuity correction<br>data: table(chisq_r2_dat$selected_2, chisq_r2_dat$misinfo_rank)<br>X-squared = 0.60915, df = 1, p-value = 0.4351 | - |
| Rank 3 | Pearson's Chi-squared test with Yates' continuity correction<br>data: table(chisq_r3_dat$selected_3, chisq_r3_dat$misinfo_rank)<br>X-squared = 1.3116, df = 1, p-value = 0.2521 | Pearson's Chi-squared test with Yates' continuity correction<br>data: table(chisq_r3_dat$selected_3, chisq_r3_dat$misinfo_rank)<br>X-squared = 2.2527, df = 1, p-value = 0.1334 | - |
| Rank 4 | Pearson's Chi-squared test with Yates' continuity correction<br>data: table(chisq_r4_dat$selected_4, chisq_r4_dat$misinfo_rank)<br>X-squared = 0.279, df = 1, p-value = 0.5974 | Pearson's Chi-squared test with Yates' continuity correction<br>data: table(chisq_r4_dat$selected_4, chisq_r4_dat$misinfo_rank)<br>X-squared = 0.087148, df = 1, p-value = 0.7678 | Pearson's Chi-squared test<br>data: table(s3_chisq_df$full_condition, s3_chisq_df$selected_4)<br>X-squared = 3.905, df = 5, p-value = 0.5632 |

Figure S24: Chi-Square tests of clicks on accurate results at positions 2, 3, and 4 in each experiment

The click probability for Result 2 in Experiment 1 is not significant at our Bonferroni-adjusted level, though it is significant at the 0.05 level. We explored this finding and observed that it was actually in the opposite direction of our hypothesis: misinformation present in the first rank *increased* the probability of participants clicking on the accurate result ranked 2nd (Figure S25). This finding makes sense – given that participants avoided clicking on misinformation, they were forced to click more often on the second result since it was the first accurate result shown.

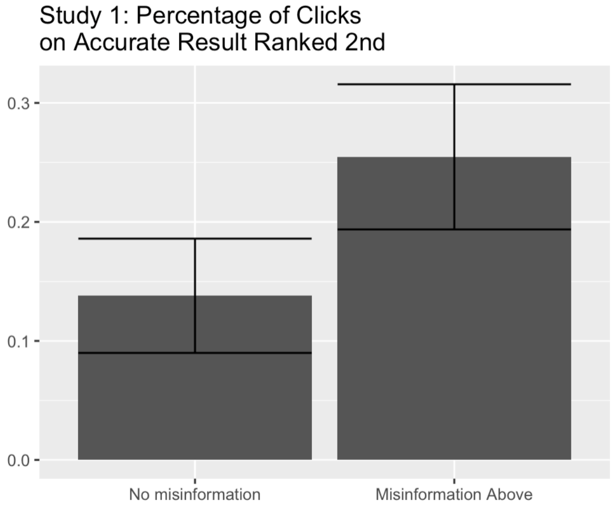

Figure S25: The clicks on an accurate result in rank 2 increased in Experiment 1 when misinformation was in rank 1 compared to when it was not present.

For Experiment 1 and Experiment 2, we had pre-registered doing chi-square tests to measure whether any of the top four accurate results' probabilities of being clicked changed between any of the possible ranks misinformation could take (and when it was absent). Our pre-registered hypothesis for this experiment was that accurate information would be more likely to be clicked when it preceded misinformation than when it followed misinformation, which we were able to test by comparing clicks on the accurate result ranked 2nd between when misinformation was ranked 1st and when misinformation was ranked 3rd. To reduce the chance of committing Type I Error, we used a Bonferroni-adjusted p-value of 0.0125 for Experiment 1 and Experiment 2 (since we were testing three different ranks of results). We ran these tests and still found no evidence that the presence of misinformation changed the likelihood of clicking an accurate result above or below it (Figure S27).

| | Experiment 1 | Experiment 2 |
|---|---|---|
| Rank 1 | Pearson's Chi-squared test<br>data: table(chisq_r1_dat$selected_1, chisq_r1_dat$misinfo_rank)<br>X-squared = 6.0412, df = 2, p-value = 0.04877 | Pearson's Chi-squared test<br>data: table(chisq_r1_dat$selected_1, chisq_r1_dat$misinfo_rank)<br>X-squared = 2.4382, df = 2, p-value = 0.2955 |
| Rank 2 | Pearson's Chi-squared test<br>data: table(chisq_r2_dat$selected_2, chisq_r2_dat$misinfo_rank)<br>X-squared = 6.3991, df = 2, p-value = 0.04078 | Pearson's Chi-squared test<br>data: table(chisq_r2_dat$selected_2, chisq_r2_dat$misinfo_rank)<br>X-squared = 0.84347, df = 2, p-value = 0.6559 |
| Rank 3 | Pearson's Chi-squared test<br>data: table(chisq_r3_dat$misinfo_rank, chisq_r3_dat$selected_3)<br>X-squared = 2.4551, df = 2, p-value = 0.293 | Pearson's Chi-squared test<br>data: table(chisq_r3_dat$misinfo_rank, chisq_r3_dat$selected_3)<br>X-squared = 6.9337, df = 2, p-value = 0.03122 |
| Rank 4 | Pearson's Chi-squared test<br>data: table(s1_chisq_df$misinfo_rank, s1_chisq_df$selected_4)<br>X-squared = 1.9257, df = 3, p-value = 0.588 | Pearson's Chi-squared test<br>data: table(s2_chisq_df$misinfo_rank, s2_chisq_df$selected_4)<br>X-squared = 1.6588, df = 3, p-value = 0.6461 |

Figure S26: Chi-Square tests of clicks on accurate results at positions 1, 2, 3, and 4 depending on rank of misinformation in Experiments 1 and 2

Although none of the tests is significant at our Bonferroni-adjusted alpha level, the tests for results ranked 1st and 2nd in Experiment 1 and the result ranked 3rd in Experiment 2 are significant at the 0.05 alpha level. We explored these cases and found that the pairwise contrasts were only significant for the result ranked 3rd when misinformation went from rank 1 to rank 2 in Experiment 2 (Figure S28). When plotting the probabilities of clicking on this result in these conditions, we found that this difference was in the opposite direction of our hypothesis: misinformation present right above *increased* the probability of participants clicking on the accurate result compared to when the misinformation was higher on the page (Figure S27). This finding again makes sense, since the presence of misinformation right above evidently pushed participants to click the next accurate result they saw.

Experiment 1, accurate result ranked 1st:

| | Comparison | p.Chisq | p.adj.Chisq |
|---|---|---|---|
| 1 | 0 : 2 | 0.1610 | 0.4830 |
| 2 | 0 : 3 | 0.5960 | 1.0000 |
| 3 | 2 : 3 | 0.0194 | 0.0582 |

Experiment 1, accurate result ranked 2nd:

| | Comparison | p.Chisq | p.adj.Chisq |
|---|---|---|---|
| 1 | 0 : 1 | 0.0375 | 0.1120 |
| 2 | 0 : 3 | 0.0228 | 0.0684 |
| 3 | 1 : 3 | 1.0000 | 1.0000 |

Experiment 2, accurate result ranked 3rd:

| | Comparison | p.Chisq | p.adj.Chisq |
|---|---|---|---|
| 1 | 0 : 1 | 0.4340 | 1.0000 |
| 2 | 0 : 2 | 0.1330 | 0.3990 |
| 3 | 1 : 2 | 0.0156 | 0.0468 |

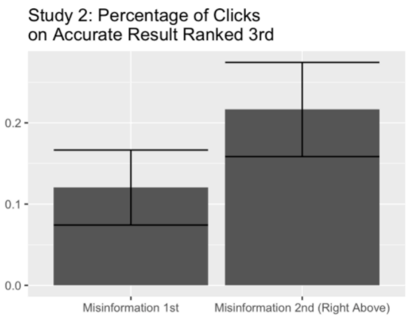


Figure S27: Misinformation does not decrease click probabilities for accurate results above or below it in Experiments 1 and 2.

We had also pre-registered a 2-Sample Kolmogorov-Smirnov test for Experiment 1 and Experiment 2. This was intended to measure whether the overall distributions of clicks on the top four results changed between misinformation being ranked 2nd and 3rd; however, this test was not appropriate for the research question since it could not control for rank. We still ran this test and it was nonsignificant in both experiments (Figure S28).

| Experiment 1 | Experiment 2 |
| --- | --- |

```
        Two-sample Kolmogorov-Smirnov test

data:  s1_mis2_clicks and s1_mis3_clicks
D = 0.66667, p-value = 0.6
alternative hypothesis: two-sided
```

```
        Two-sample Kolmogorov-Smirnov test

data:  s2_mis2_clicks and s2_mis3_clicks
D = 1, p-value = 0.1
alternative hypothesis: two-sided
```

Figure S28: Pre-registered Kolmogorov-Smirnov tests of clicks on the three topmost accurate results when misinformation was ranked 2nd versus 3rd

Finally, for Experiment 3 we pre-registered the hypothesis that an accurate result in the same rank was more likely to be clicked when presented above a result with misinformation compared to when no misinformation was shown. To do this, we performed chi-square tests on accurate results in rank 1 and 2 for each combination of misinformation and warning condition (the same procedure used to test for downstream effects of misinformation on click likelihood for the accurate result in rank 4). None of these tests showed any differences in the probability of clicking on an accurate result based on misinformation's presence below it (Figure S29).

Experiment 3, accurate result ranked 1st:

```
        Pearson's Chi-squared test

data:  table(s3_chisq_df$full_condition, s3_chisq_df$selected_1)
X-squared = 0.85809, df = 2, p-value = 0.6511
```

Experiment 3, accurate result ranked 2nd:

```
        Pearson's Chi-squared test

data:  table(s3_chisq_df$full_condition, s3_chisq_df$selected_2)
X-squared = 2.6628, df = 2, p-value = 0.2641
```

Figure S29: Accurate results above misinformation are no more likely to be clicked than when no misinformation is present in Experiment 3.

**Clicking, information quality, and warnings' associations with trust:** In Experiment 1, we measured the association between clicking and information quality with trust levels using repeated measures ANOVAs due to participants rating multiple results covering these distinctions. Due to these factors not being fully crossed, we had to run two separate ANOVAs for each factor on subsets of the data for which we had multiple ratings from each participant. We checked the effect sizes of these models against those found in the full linear regression with rank included (see following section for full regression table) and found that they were within hundredths of each other and remained significant (Figures S30 and S33).

Experiment 1: Clicking

```
Univariate Type III Repeated-Measures ANOVA Assuming Sphericity

             Sum Sq num Df Error SS den Df  F value    Pr(>F)
(Intercept)  23494.8      1  1617.21    979 14222.93 < 2.2e-16 ***
Click_status   245.7      1   522.27    979   460.63 < 2.2e-16 ***
---
Signif. codes:  0 '***' 0.001 '**' 0.01 '*' 0.05 '.' 0.1 ' ' 1


P value adjustment: bonferroni method for 2 tests

$contrasts
 contrast                            estimate    SE  df t.ratio p.value
 Clicked.result - Non.clicked.result    0.708 0.033 979  21.462  <.0001
```

Experiment 1: Information quality

```
Univariate Type III Repeated-Measures ANOVA Assuming Sphericity

             Sum Sq num Df Error SS den Df F value    Pr(>F)
(Intercept)  8003.5      1   788.52    781  7927.2 < 2.2e-16 ***
Info_quality 1134.4      1   723.59    781  1224.4 < 2.2e-16 ***
---
Signif. codes:  0 '***' 0.001 '**' 0.01 '*' 0.05 '.' 0.1 ' ' 1


P value adjustment: bonferroni method for 2 tests

$contrasts
 contrast                               estimate     SE  df t.ratio p.value
 Accurate.Information - Misinformation       1.7 0.0487 781  34.992  <.0001
```

Figure S30: Two repeated-measures ANOVAs for differences in trust depending on clicking and quality in Experiment 1

In Experiment 2 and Experiment 3, participants only rated one result (instead of three), so we calculated the effects of clicking and information quality using normal ANOVAs on the full dataset. In the main text, we report on the estimates when rank was not included in this model because we had pre-registered this analysis as its own test; note that including rank did not significantly change these estimates (Figures S31-S33).

```
Anova Table (Type II tests)

Response: Trust_ratings_of_results
              Sum Sq  Df F value    Pr(>F)
Info_quality  130.01   1 111.199 < 2.2e-16 ***
Click_status   68.62   1  58.689  4.37e-14 ***
Residuals    1163.33 995
---
Signif. codes:  0 ‘***’ 0.001 ‘**’ 0.01 ‘*’ 0.05 ‘.’ 0.1 ‘ ’ 1
```

```
Results are averaged over the levels of: Click_status
P value adjustment: bonferroni method for 2 tests

$contrasts
 contrast                              estimate     SE  df t.ratio p.value
 Accurate Information - Misinformation    0.911 0.0864 995  10.545  <.0001
```

```
Results are averaged over the levels of: Info_quality
P value adjustment: bonferroni method for 2 tests

$contrasts
 contrast                              estimate     SE  df t.ratio p.value
 Clicked result - (Non-clicked result)    0.594 0.0775 995   7.661  <.0001
```

Figure S31: Between-subjects ANOVA and pairwise contrasts for differences in trust depending on clicking and quality in Experiment 2.

In Experiment 3, we added the Warning condition as a predictor to this model. Given that we had pre-registered measuring a 3-way interaction between warnings, clicking, and misinformation in this experiment but had insufficient statistical power to do this test as planned due to the lack of people who clicked on misinformation, we ran the model with two 2-way interactions (one between clicking and warning condition, the other between information quality and warning condition). While neither interaction term was significant, post-hoc contrasts using this model showed that the main effect of the Source Reputation Warning was driven by the ratings for accurate results alone, whereas it showed no effect for misinformation results.

That said, we noticed that trust in the misinformation dropped similarly under this warning condition (difference in means = 0.19 scale points). We performed a post-hoc power analysis on the number of ratings necessary to have 80% statistical power to detect this effect at significance and found that we did not have enough ratings to do so. We used the “pwr2ppl” package in R to conduct this analysis on a t-test, with Cohen’s $d = 0.19$ (the resulting effect size from the linear model) with desired power = 0.80 and alpha = 0.05. The analysis showed that we would have needed 430 ratings of the misinformation results per warning condition to detect this effect; we only had 50-54 ratings per warning condition, so this may be a significant decrease in reality.

```
Anova Table (Type II tests)

Response: Trust_ratings_of_results
                                Sum Sq   Df F value    Pr(>F)    
Info_quality                     66.56    1 54.3251 3.169e-13 ***
Warning_condition                11.44    2  4.6667  0.009576 ** 
Click_status                     95.71    1 78.1175 < 2.2e-16 ***
Info_quality:Warning_condition    0.13    2  0.0512  0.950068    
Warning_condition:Click_status    1.67    2  0.6804  0.506596    
Residuals                      1459.24 1191
```

```
Anova Table (Type III tests)

Response: Trust_ratings_of_results
                                Sum Sq   Df   F value    Pr(>F)    
(Intercept)                    1295.87    1 1057.6575 < 2.2e-16 ***
Info_quality                     26.26    1   21.4327 4.066e-06 ***
Warning_condition                 4.87    2    1.9860    0.1377    
Click_status                     38.43    1   31.3668 2.651e-08 ***
Info_quality:Warning_condition    0.13    2    0.0512    0.9501    
Warning_condition:Click_status    1.67    2    0.6804    0.5066    
Residuals                      1459.24 1191                        
---
Signif. codes:  0 ‘***’ 0.001 ‘**’ 0.01 ‘*’ 0.05 ‘.’ 0.1 ‘ ’ 1
```

```
Results are averaged over the levels of: Info_quality, Warning_condition
P value adjustment: bonferroni method for 2 tests

$contrasts
 contrast                              estimate    SE   df t.ratio p.value
 Clicked result - (Non-clicked result)    0.611 0.069 1191   8.853  <.0001
```

```
Results are averaged over the levels of: Warning_condition, Click_status
P value adjustment: bonferroni method for 2 tests

$contrasts
 contrast                              estimate     SE   df t.ratio p.value
 Accurate Information - Misinformation    0.732 0.0996 1191   7.352  <.0001
```

```
$contrasts
Info_quality = Accurate information:
 contrast                                                estimate     SE   df t.ratio p.value
 No warning - Evolving Information Warning                 0.1040 0.0849 1191   1.224  0.6631
 No warning - Source Reputation Warning                    0.2448 0.0856 1191   2.858  0.0130
 Evolving Information Warning - Source Reputation Warning  0.1408 0.0845 1191   1.666  0.2880

Info_quality = Misinformation:
 contrast                                                estimate     SE   df t.ratio p.value
 No warning - Evolving Information Warning                 0.0326 0.2320 1191   0.140  1.0000
 No warning - Source Reputation Warning                    0.1839 0.2315 1191   0.794  1.0000
 Evolving Information Warning - Source Reputation Warning  0.1514 0.2363 1191   0.641  1.0000

Results are averaged over the levels of: Click_status
P value adjustment: bonferroni method for 3 tests
```

Figure S32: ANOVAs testing for main effects of and interactions between clicking, quality, and warning interactions in Experiment 3

**Rank effect on trust:** To evaluate whether ranking of search results had an impact on their perceived trustworthiness, we added the results’ ranking as a predictor to the linear models used to test the clicking, quality, and warning effects above. None of the coefficients for rank in any of the experiments were significant (Figure S33). Note that we had forgotten to include click status as a control variable in the model for Experiment 1 in our pre-registration, but this was necessary due to our design limiting the ranks of non-clicked results participants rated to the top five, which would cause a spurious rank-trust relationship due to people trusting results they did not click less than ones they did. This control was unnecessary for the other two experiments, but we include it in the models for each below to show that trust’s association with clicking remained when result rank was added to these models.

| | *Dependent variable:* | | |
|---|---|---|---|
| | Trust_ratings_of_results | | |
| | (1) | (2) | (3) |
| Result_rank | 0.001 | 0.015 | 0.014 |
| | (0.010) | (0.013) | (0.012) |
| Info_qualityMisinformation | -1.643*** | -0.870*** | -0.704*** |
| | (0.047) | (0.093) | (0.102) |
| Click_statusNon-clicked result | -0.757*** | -0.597*** | -0.620*** |
| | (0.045) | (0.078) | (0.070) |
| Warning_conditionEvolving Information Warning | | | -0.072 |
| | | | (0.078) |
| Warning_conditionSource Reputation Warning | | | -0.237*** |
| | | | (0.078) |
| Constant | 3.826*** | 3.091*** | 2.949*** |
| | (0.054) | (0.085) | (0.085) |
| Observations | 2,994 | 998 | 1,200 |
| $R^2$ | 0.455 | 0.203 | 0.139 |
| Adjusted $R^2$ | 0.454 | 0.200 | 0.135 |
| Residual Std. Error | 1.019 (df = 2990) | 1.081 (df = 994) | 1.106 (df = 1194) |
| F Statistic | 830.516*** (df = 3; 2990) | 84.152*** (df = 3; 994) | 38.570*** (df = 5; 1194) |
| *Note:* | | | *p<0.1; **p<0.05; ***p<0.01 |

Figure S33: Reported linear regressions of rank, clicking, quality, and warning effects on trust without interaction terms (all experiments)

To better match the pre-registered models (which included 3-way interactions that were infeasible due to statistical power limitations), we ran versions of these models with 2-way interactions (rank with clicking in all three experiments, rank with information quality in Experiment 1 and Experiment 2, and rank with warning condition in Experiment 3), allowing us to evaluate whether ranking had different effects on trustworthiness appraisals for any of these specific subsets of search results. None of these interactions was significant, providing support for the overall null effect of ranking on trustworthiness judgments (Figures S34-S35).

Experiment 1

```
Anova Table (Type III tests)

Response: Trust_ratings_of_results
                          Sum Sq   Df   F value Pr(>F)
(Intercept)               4194.5    1 4037.1594 <2e-16 ***
Result_rank                  0.0    1    0.0073 0.9318
Info_quality               172.3    1  165.8065 <2e-16 ***
Click_status                75.6    1   72.7554 <2e-16 ***
Result_rank:Info_quality     0.1    1    0.0602 0.8062
Result_rank:Click_status     0.2    1    0.1666 0.6831
Residuals                 3104.4 2988
---
Signif. codes:  0 ‘***’ 0.001 ‘**’ 0.01 ‘*’ 0.05 ‘.’ 0.1 ‘ ’ [illegible]
```

Experiment 2

```
Anova Table (Type III tests)

Response: Trust_ratings_of_results
                          Sum Sq  Df  F value    Pr(>F)
(Intercept)               894.73   1 766.1345 < 2.2e-16 ***
Result_rank                 0.28   1   0.2364 0.6269183
Info_quality               12.77   1  10.9374 0.0009762 ***
Click_status               34.32   1  29.3850 7.452e-08 ***
Result_rank:Info_quality    0.59   1   0.5086 0.4759311
Result_rank:Click_status    2.84   1   2.4287 0.1194480
Residuals                1158.50 992
---
Signif. codes:  0 ‘***’ 0.001 ‘**’ 0.01 ‘*’ 0.05 ‘.’ 0.1 ‘ ’ 1
```

Experiment 3

```
Anova Table (Type III tests)

Response: Trust_ratings_of_results
                               Sum Sq   Df  F value    Pr(>F)
(Intercept)                    624.77    1 511.8352 < 2.2e-16 ***
Result_rank                      1.44    1   1.1800    0.2776
Click_status                    34.01    1  27.8645 1.545e-07 ***
Warning_condition                0.24    2   0.0993    0.9055
Info_quality                    55.05    1  45.1010 2.891e-11 ***
Result_rank:Click_status         0.22    1   0.1775    0.6736
Result_rank:Warning_condition    5.62    2   2.3010    0.1006
Residuals                     1453.80 1191
---
Signif. codes:  0 ‘***’ 0.001 ‘**’ 0.01 ‘*’ 0.05 ‘.’ 0.1 ‘ ’ 1
```

Figure S34: Type III ANCOVAs finding no interactions between rank and the other factors (all experiments). Note: there is no term for a rank-by-quality interaction in the model for Experiment 3 because the misinformation result could only appear in one rank for this experiment

For Experiment 3, we had preregistered running this model only on the accurate results (with no information quality predictor) and with a 3-way interaction term between rank, click status, and warning condition. We did this analysis and found the same main effects of clicking and warning from the regression above, with no significant interactions between rank and the other factors (Figure S35).

```
Anova Table (Type II tests)

Response: Trust_ratings_of_results
                                          Sum Sq   Df F value  Pr(>F)
Result_rank                                 1.53    1  1.2339 0.26691
Click_status                               98.03    1 79.1817 < 2e-16 ***
Warning_condition                          10.58    2  4.2708 0.01422 *
Result_rank:Click_status                    0.28    1  0.2223 0.63738
Result_rank:Warning_condition               5.51    2  2.2254 0.10854
Click_status:Warning_condition              1.17    2  0.4727 0.62348
Result_rank:Click_status:Warning_condition  5.35    2  2.1586 0.11600
Residuals                                1280.19 1034
---
Signif. codes:  0 '***' 0.001 '**' 0.01 '*' 0.05 '.' 0.1 ' ' 1
```

```
Anova Table (Type III tests)

Response: Trust_ratings_of_results
                                          Sum Sq   Df  F value    Pr(>F)
(Intercept)                               408.28    1 329.7649 < 2.2e-16 ***
Result_rank                                 0.24    1   0.1911   0.66213
Click_status                               25.30    1  20.4367 6.875e-06 ***
Warning_condition                           5.14    2   2.0749   0.12609
Result_rank:Click_status                    3.84    1   3.1036   0.07841 .
Result_rank:Warning_condition               4.82    2   1.9475   0.14315
Click_status:Warning_condition              6.13    2   2.4751   0.08466 .
Result_rank:Click_status:Warning_condition  5.35    2   2.1586   0.11600
Residuals                                1280.19 1034
---
Signif. codes:  0 '***' 0.001 '**' 0.01 '*' 0.05 '.' 0.1 ' ' 1
```

Figure S35: Type II and Type III ANCOVAs checking for consistency of results in pre-registered model (on accurate results only) for Experiment 3.

**Misinformation's null effect on trust in accurate results:** To measure the downstream effect of a result with misinformation on participants' trust in accurate information below it, we focused on comparing trust ratings of accurate results that appeared immediately below a misinformation result in one of the treatment groups with its ratings in the control group (in which no misinformation was present on the page). This meant that in Experiment 1 and Experiment 2, we looked at the results in ranks 2, 3, and 4; in Experiment 3, we only looked at the 4th result since we presented it in rank 3, only. We used an ANCOVA model to control for rank and whether the participant was rating the result they clicked or one they had not clicked. In all experiments, we found no effect of seeing misinformation immediately above an accurate result on participants' levels of trust in the accurate result (Figure S36-S37). Type III ANCOVAs showed no interactions with the result's rank, whether people had clicked the result, or the presence of a warning (Figure S37).

| | *Dependent variable:* | | |
|---|---|---|---|
| | Trust_ratings_of_results | | |
| | (1) | (2) | (3) |
| mis_presentMisinformation right above | 0.053 | 0.237 | -0.050 |
| | (0.088) | (0.166) | (0.215) |
| Result_rank | -0.046 | 0.117 | |
| | (0.053) | (0.094) | |
| Click_statusNon-clicked result | $-0.602^{***}$ | $-0.921^{***}$ | $-0.561^{***}$ |
| | (0.094) | (0.168) | (0.212) |
| Warning_conditionEvolving Information Warning | | | 0.043 |
| | | | (0.265) |
| Warning_conditionSource Reputation Warning | | | -0.268 |
| | | | (0.245) |
| Constant | $3.878^{***}$ | $2.759^{***}$ | $2.970^{***}$ |
| | (0.176) | (0.301) | (0.233) |
| Observations | 643 | 129 | 102 |
| $R^2$ | 0.065 | 0.197 | 0.091 |
| Adjusted $R^2$ | 0.060 | 0.178 | 0.053 |
| Residual Std. Error | 1.086 (df = 639) | 0.919 (df = 125) | 1.048 (df = 97) |
| F Statistic | $14.726^{***}$ (df = 3; 639) | $10.251^{***}$ (df = 3; 125) | $2.415^{*}$ (df = 4; 97) |
| *Note:* | | | $^{*}$p<0.1; $^{**}$p<0.05; $^{***}$p<0.01 |

Figure S36: Reported linear models showing no effect of misinformation on trust in accurate information directly below it (all experiments).

Experiment 1

```
Anova Table (Type III tests)

Response: Trust_ratings_of_results
                          Sum Sq  Df  F value    Pr(>F)    
(Intercept)               290.68   1 246.1866 < 2.2e-16 ***
mis_present                 0.74   1   0.6278    0.4285    
Result_rank                 1.77   1   1.5021    0.2208    
Click_status               25.95   1  21.9787 3.374e-06 ***
mis_present:Result_rank     0.81   1   0.6829    0.4089    
mis_present:Click_status    0.30   1   0.2521    0.6157    
Residuals                 752.12 637                       
---
Signif. codes:  0 ‘***’ 0.001 ‘**’ 0.01 ‘*’ 0.05 ‘.’ 0.1 ‘ ’ 1
```

Experiment 2

```
Anova Table (Type III tests)

Response: Trust_ratings_of_results
                          Sum Sq  Df F value    Pr(>F)    
(Intercept)               28.798   1 33.9663 4.611e-08 ***
mis_present                1.983   1  2.3383  0.128793    
Result_rank                2.632   1  3.1040  0.080585 .  
Click_status              12.584   1 14.8429  0.000187 ***
mis_present:Result_rank    1.308   1  1.5429  0.216551    
mis_present:Click_status   0.056   1  0.0662  0.797317    
Residuals                104.284 123                      
---
Signif. codes:  0 ‘***’ 0.001 ‘**’ 0.01 ‘*’ 0.05 ‘.’ 0.1 ‘ ’ 1
```

Experiment 3

```
Anova Table (Type III tests)

Response: Trust_ratings_of_results
                               Sum Sq Df  F value Pr(>F)    
(Intercept)                   125.469  1 112.6346 <2e-16 ***
mis_present                     0.439  1   0.3945 0.5315    
Click_status                    6.867  1   6.1649 0.0148 *  
Warning_condition               0.476  2   0.2138 0.8079    
mis_present:Click_status        0.816  1   0.7324 0.3943    
mis_present:Warning_condition   1.251  2   0.5617 0.5721    
Residuals                     104.711 94                    
---
Signif. codes:  0 ‘***’ 0.001 ‘**’ 0.01 ‘*’ 0.05 ‘.’ 0.1 ‘ ’ 1
```

Figure S37: Type III ANCOVAs testing for interactions between misinformation presence/position and the other factors on trust in accurate result directly below (all experiments)

We had pre-registered testing the impacts of misinformation presence on people's trust in accurate results above the misinformation, as well as all of the accurate results in the page. We also preregistered testing how trust was affected depending on the relative position of the misinformation to accurate results (whether it was shown *above or below* them). We ran these models and still found no effects of exposure to misinformation on participants' trust in accurate information (Figures S38-S41).

| | *Dependent variable:* | | |
|---|---|---|---|
| | Trust_ratings_of_results | | |
| | (1) | (2) | (3) |
| Exposed_to_misinformationnot exposed | 0.067 | -0.271 | 0.144 |
| | (0.088) | (0.183) | (0.122) |
| Click_statusNon-clicked result | $-0.767^{***}$ | $-0.697^{***}$ | $-0.583^{***}$ |
| | (0.084) | (0.166) | (0.121) |
| Constant | $3.785^{***}$ | $3.195^{***}$ | $2.784^{***}$ |
| | (0.066) | (0.149) | (0.112) |
| Observations | 656 | 227 | 369 |
| $R^2$ | 0.115 | 0.073 | 0.081 |
| Adjusted $R^2$ | 0.112 | 0.065 | 0.076 |
| Residual Std. Error | 1.044 (df = 653) | 1.128 (df = 224) | 1.090 (df = 366) |
| F Statistic | $42.317^{***}$ (df = 2; 653) | $8.846^{***}$ (df = 2; 224) | $16.165^{***}$ (df = 2; 366) |
| *Note:* | | | $^{*}$p<0.1; $^{**}$p<0.05; $^{***}$p<0.01 |

Figure S38: No effect of the mere presence of misinformation on trust ratings of accurate results above it on the page (compared to same-ranked accurate results when no misinformation was shown).

| | *Dependent variable:* | | |
|---|---|---|---|
| | Trust_ratings_of_results | | |
| | (1) | (2) | (3) |
| Exposed_to_misinformationnot exposed | 0.017 | -0.059 | 0.050 |
| | (0.052) | (0.090) | (0.071) |
| Click_statusNon-clicked result | -0.735*** | -0.580*** | -0.600*** |
| | (0.046) | (0.080) | (0.071) |
| Constant | 3.813*** | 3.168*** | 2.869*** |
| | (0.036) | (0.067) | (0.071) |
| Observations | 2,194 | 770 | 1,046 |
| $R^2$ | 0.104 | 0.065 | 0.068 |
| Adjusted $R^2$ | 0.103 | 0.062 | 0.066 |
| Residual Std. Error | 1.071 (df = 2191) | 1.080 (df = 767) | 1.118 (df = 1043) |
| F Statistic | 127.091*** (df = 2; 2191) | 26.447*** (df = 2; 767) | 37.792*** (df = 2; 1043) |
| *Note:* | | | *p<0.1; **p<0.05; ***p<0.01 |

Figure S39: No effect of the mere presence of misinformation on trust ratings of all accurate results on the page.

| | *Dependent variable:* | | |
|---|---|---|---|
| | Trust_ratings_of_results | | |
| | (1) | (2) | (3) |
| mis_positionMisinformation Below | -0.079 | -0.007 | -0.117 |
| | (0.076) | (0.132) | (0.130) |
| mis_positionNone present | -0.006 | -0.064 | -0.007 |
| | (0.057) | (0.096) | (0.089) |
| Result_rank | -0.005 | 0.015 | 0.005 |
| | (0.013) | (0.017) | (0.015) |
| Click_statusNon-clicked result | $-0.741^{***}$ | $-0.583^{***}$ | $-0.604^{***}$ |
| | (0.049) | (0.081) | (0.072) |
| Warning_conditionEvolving Information Warning | | | -0.094 |
| | | | (0.085) |
| Warning_conditionSource Reputation Warning | | | $-0.246^{***}$ |
| | | | (0.085) |
| Constant | $3.859^{***}$ | $3.097^{***}$ | $3.016^{***}$ |
| | (0.083) | (0.122) | (0.133) |
| Observations | 2,194 | 770 | 1,046 |
| $R^2$ | 0.104 | 0.066 | 0.077 |
| Adjusted $R^2$ | 0.103 | 0.062 | 0.071 |
| Residual Std. Error | 1.071 (df = 2189) | 1.080 (df = 765) | 1.115 (df = 1039) |
| F Statistic | $63.818^{***}$ (df = 4; 2189) | $13.611^{***}$ (df = 4; 765) | $14.408^{***}$ (df = 6; 1039) |
| *Note:* | | | $^{*}$p<0.1; $^{**}$p<0.05; $^{***}$p<0.01 |

Figure S40: No effect of misinformation's relative position on trust in accurate information across the entire page.

Experiment 1

```
Anova Table (Type III tests)

Response: Trust_ratings_of_results
                            Sum Sq   Df   F value Pr(>F)
(Intercept)                1368.72    1 1190.5814 <2e-16 ***
mis_position                  0.08    2    0.0362 0.9645
Result_rank                   0.07    1    0.0645 0.7996
Click_status                124.47    1  108.2692 <2e-16 ***
mis_position:Result_rank      0.02    2    0.0068 0.9932
mis_position:Click_status     0.10    2    0.0436 0.9574
Residuals                  2511.92 2185
---
Signif. codes:  0 '***' 0.001 '**' 0.01 '*' 0.05 '.' 0.1 ' ' 1
```

Experiment 2

```
Anova Table (Type III tests)

Response: Trust_ratings_of_results
                           Sum Sq  Df  F value     Pr(>F)
(Intercept)                477.23   1 407.7475 < 2.2e-16 ***
mis_position                 1.99   2   0.8522    0.4269
Result_rank                  0.10   1   0.0882    0.7666
Click_status                33.32   1  28.4662 1.259e-07 ***
mis_position:Result_rank     0.47   2   0.1992    0.8194
mis_position:Click_status    1.30   2   0.5567    0.5733
Residuals                  890.68 761
---
Signif. codes:  0 '***' 0.001 '**' 0.01 '*' 0.05 '.' 0.1 ' ' 1
```

Experiment 3

```
Anova Table (Type III tests)

Response: Trust_ratings_of_results
                                         Sum Sq   Df F value  Pr(>F)
(Intercept)                              107.57    1 86.9054 < 2e-16 ***
mis_position                               1.97    2  0.7969 0.45102
Result_rank                                1.37    1  1.1077 0.29282
Click_status                               3.01    1  2.4309 0.11927
Warning_condition                          7.58    2  3.0627 0.04719 *
mis_position:Result_rank                   3.39    2  1.3680 0.25508
mis_position:Click_status                  1.97    2  0.7968 0.45105
mis_position:Warning_condition             7.65    4  1.5457 0.18679
Click_status:Warning_condition             1.89    2  0.7626 0.46672
mis_position:Click_status:Warning_condition 6.27   4  1.2667 0.28127
Residuals                               1268.71 1025
---
Signif. codes:  0 '***' 0.001 '**' 0.01 '*' 0.05 '.' 0.1 ' ' 1
```

Figure S41: Type III ANCOVAs showing no interactions between misinformation presence/position and the other factors in pre-registered models on all accurate results (all experiments)

## 9. Exploratory analyses

**Are there demographic moderators of any of our results?**
To ensure that our main effects remained significant when controlling for participants' demographic group membership and attitudes relevant to the tasks (political alignment, general support for vaccination, and general levels of trust in Google or DuckDuckGo), we reran the reported models testing the relationships of click likelihood and trustworthiness evaluations with these factors added as interaction terms with the manipulated factors (information quality, result rank, and misinformation presence). We re-coded vaccine supportiveness and trust in the search engine by classifying three groups of people: low group (mean scores < 2.5), moderate group (means scores between 2.5 and <= 3.5), and high group (mean scores >= 3.5). For political views, we classified based on the integer scale ratings (< 3 = conservative, 3 = moderate, > 3 = liberal). We used the summary() command to see whether any of these moderator variables were significant.

None of our treatment (non-)effects ~~as reported in the main text~~ changed when controlling for these participant-level factors, except for three cases outlined here:

1. Probability of clicking on result depending on its quality: participants' general support for vaccination made an impact on the difference in the probability of clicking the 1st-ranked result in Experiments 1 and 2. Only participants who highly support vaccination showed a significantly lower probability of clicking on the first result when it had misinformation; people with moderate or low support for vaccination showed no significant differences in propensities to click the result regardless of its quality (Figure S42). This finding did not replicate for the 2nd and 3rd ranked results, nor in Experiment 3.

2. Effects of information quality on trust in results:

    a. In Experiment 1: people who were low or moderately supportive of vaccines did not show significantly lower trust in misinformation than accurate information.

    b. In Experiment 2: people who had low or moderate support for vaccines didn't show significant differences in trust toward accurate and misinformation results; only high supporters did.

    c. In Experiment 3: Political moderates and conservatives did not show significant differences in trust toward accurate and misinformation results, only liberals did.

3. Effects of warning banner on trust in accurate results: the "source reputation" warning's decreased trust in accurate results was only significant for people who had high support in vaccination (the majority of the sample).

4. Effects of misinformation's presence on trust in all accurate results on the same page: In Experiment 3 only, people who had low trust in the search engine actually showed *increased* trust in accurate results when they were exposed to

misinformation in the list compared to when they weren't (Figure S43). This could be because people who are normally distrustful of search engines become more confident in their ability to discern what's accurate when they see a blatantly false result.

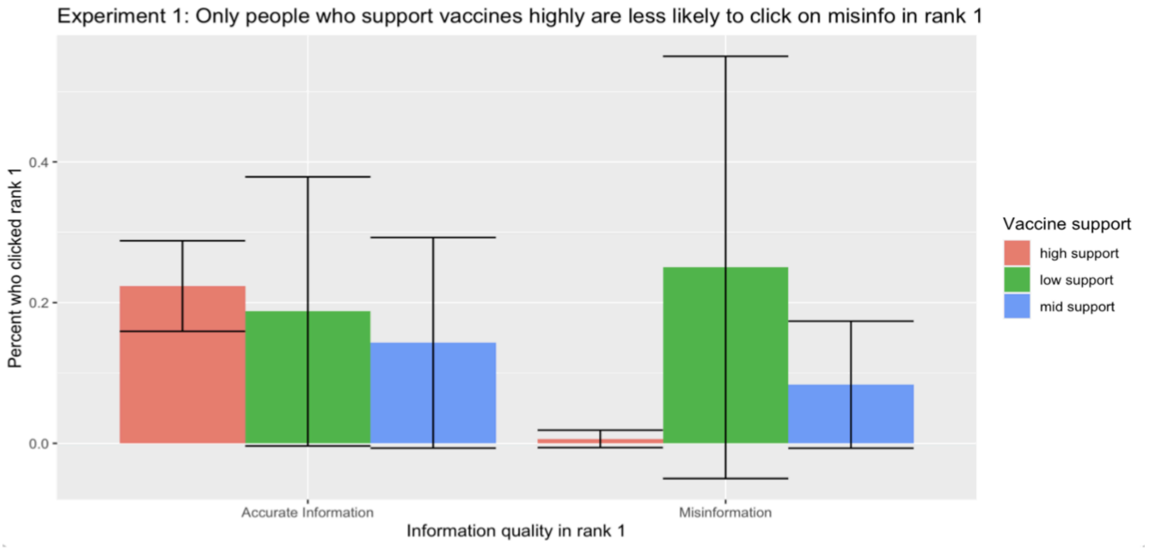


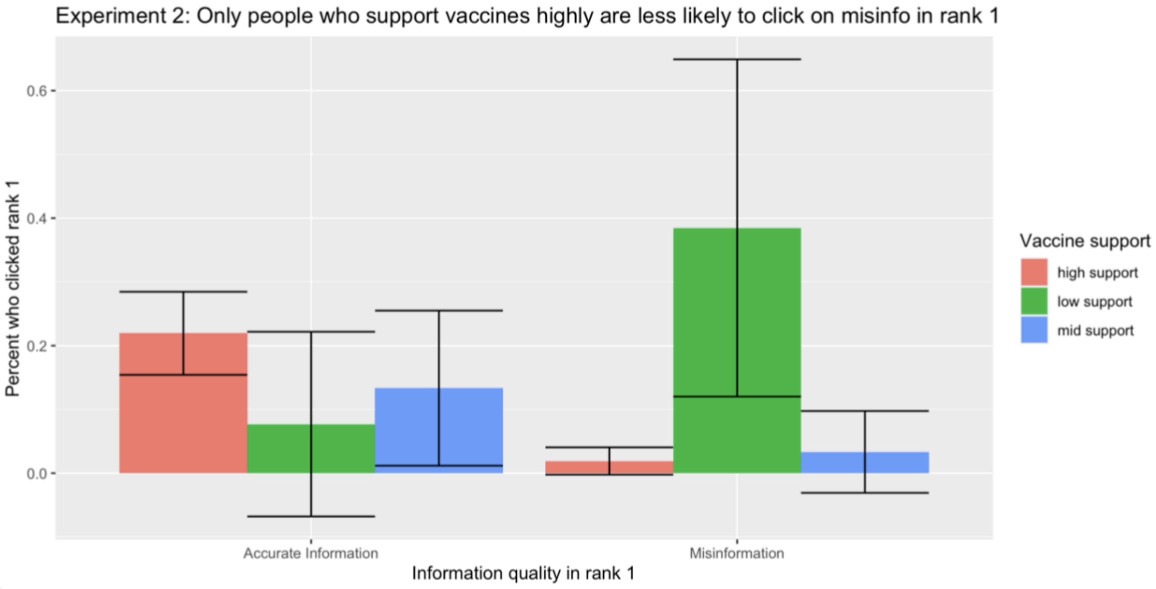


Figure S42: Only people with high support for vaccines were significantly less likely to click on top-ranked result when it was misinformation in Experiments 1-2.

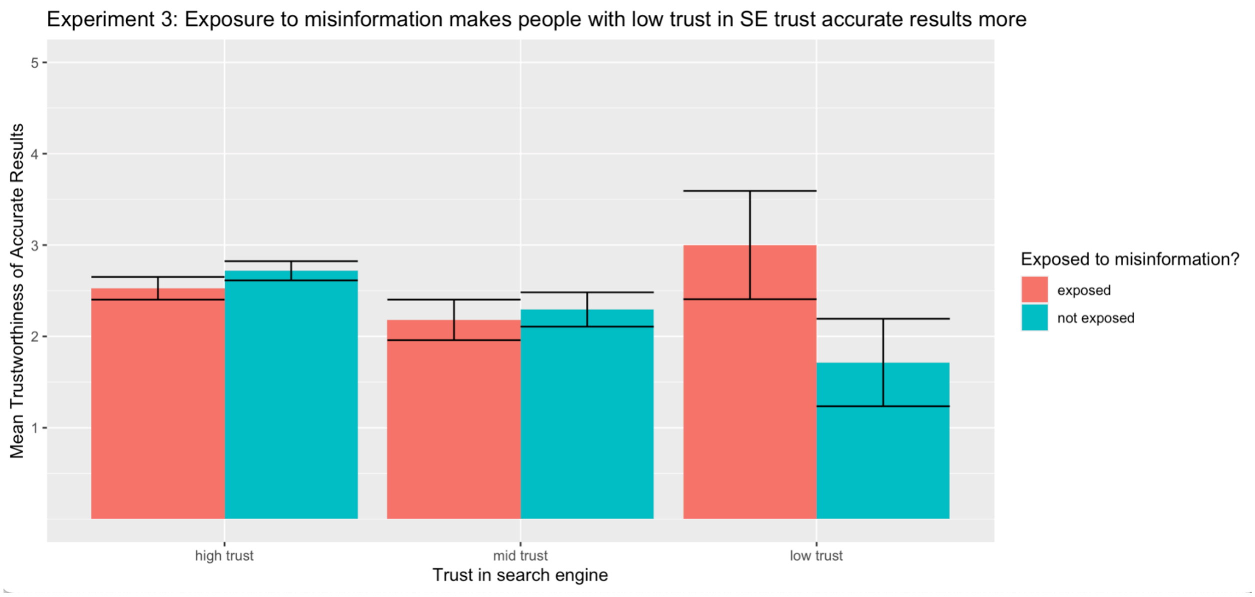


Figure S43. People in Experiment 3 who distrust search engines experienced increased trust in accurate results when shown a misinformation result on the same page.

**Does rank impact the perceived relevance of search results' information?**

We ran models to test whether rank impacted relevance ratings of the results (controlling for click status and their information quality), and found null effects in each experiment (Fig. S44).

Experiment 1

```
Coefficients:
                              Estimate Std. Error t value Pr(>|t|)
(Intercept)                    4.27285    0.05754  74.262   <2e-16 ***
Result_rank                   -0.01242    0.01020  -1.217    0.224
Info_qualityMisinformation    -1.45951    0.04975 -29.339   <2e-16 ***
Click_statusNon-clicked result -0.71144   0.04728 -15.047   <2e-16 ***
---
Signif. codes:  0 ‘***’ 0.001 ‘**’ 0.01 ‘*’ 0.05 ‘.’ 0.1 ‘ ’ 1

Residual standard error: 1.078 on 2990 degrees of freedom
Multiple R-squared:  0.3713,    Adjusted R-squared:  0.3707
F-statistic: 588.6 on 3 and 2990 DF,  p-value: < 2.2e-16
```

Experiment 2

```
Coefficients:
                                Estimate Std. Error t value Pr(>|t|)
(Intercept)                    4.2069448  0.0890308  47.253  < 2e-16 ***
Result_rank                   -0.0008129  0.0132481  -0.061    0.951
Info_qualityMisinformation    -1.0908552  0.0977011 -11.165  < 2e-16 ***
Click_statusNon-clicked result -0.4797386 0.0811094  -5.915 4.57e-09 ***
---
Signif. codes:  0 ‘***’ 0.001 ‘**’ 0.01 ‘*’ 0.05 ‘.’ 0.1 ‘ ’ 1

Residual standard error: 1.131 on 994 degrees of freedom
Multiple R-squared:  0.2044,    Adjusted R-squared:  0.202
F-statistic: 85.14 on 3 and 994 DF,  p-value: < 2.2e-16
```

Experiment 3

```
Coefficients:
                                             Estimate Std. Error t value Pr(>|t|)
(Intercept)                                   4.16849    0.08176  50.985  < 2e-16 ***
Result_rank                                   0.01628    0.01094   1.488    0.137
Click_statusNon-clicked result               -0.48946    0.06581  -7.438 2.14e-13 ***
Warning_conditionEvolving Information Warning  0.02001    0.07875   0.254    0.800
Warning_conditionSource Reputation Warning   -0.04241    0.07893  -0.537    0.591
---
Signif. codes:  0 ‘***’ 0.001 ‘**’ 0.01 ‘*’ 0.05 ‘.’ 0.1 ‘ ’ 1

Residual standard error: 1.039 on 1041 degrees of freedom
Multiple R-squared:  0.05149,   Adjusted R-squared:  0.04784
F-statistic: 14.13 on 4 and 1041 DF,  p-value: 3.126e-11
```

Figure S44. Relevance ratings are unaffected by rank (with controls for click status, information quality, and warning condition in Experiment 3).

## Section 10. Post-hoc power analysis for warnings' effects

We only saw an effect of the "source reputation" warning for the accurate results, but the misinformation results had a similar descriptive decrease in trust between the warning conditions and we had fewer ratings of the misinformation results than of the accurate results. We did a post-hoc power analysis with the effect size of the decreased trust seen for the misinformation (d = 0.19) to see how many ratings of misinformation result would have been necessary to detect a comparable effect of the warning, if one existed. We found that we had insufficient statistical power to detect this effect: for 80% power, we would have needed ratings from at least 430 people for the misinformation per warning condition, while we only had 50-54.
